# Multi-isotopic analysis of zooarchaeological material from Estonia (ca. 200–1800 CE): Variation among food webs and geographical regions

**DOI:** 10.1371/journal.pone.0279583

**Published:** 2022-12-27

**Authors:** Ülle Aguraiuja-Lätti, Mari Tõrv, Kerry L. Sayle, Lembi Lõugas, Eve Rannamäe, Freydis Ehrlich, Sander Nuut, Taavi Peeters, Ester Oras, Aivar Kriiska

**Affiliations:** 1 Archaeological Research Collection, Tallinn University, Tallinn, Estonia; 2 Institute of History and Archaeology, University of Tartu, Tartu, Estonia; 3 Institute of Chemistry, University of Tartu, Tartu, Estonia; 4 Scottish Universities Environmental Research Centre, East Kilbride, Scotland, United Kingdom; University of Padova: Universita degli Studi di Padova, ITALY

## Abstract

To better comprehend the dietary practices of past populations in the Eastern Baltic region we have created temporally and geographically restricted baselines for the time period of 200–1800 CE. In this multi-isotopic analysis, we report new δ^13^C, δ^15^N and δ^34^S values for 251 faunal bone collagen samples from various archaeological contexts in Estonia representing the most comprehensive set of Iron Age, Medieval and Early Modern Period faunal stable isotope values to date. The results map out the local carbon and nitrogen baselines and define isotopic ranges of local terrestrial, avian and aquatic fauna. We also demonstrate the potential application of sulfur stable isotope analysis in archaeological research. The results demonstrate a clear distinction between δ^13^C and δ^34^S values of marine and terrestrial species, however, freshwater fish display notable overlaps with both marine and terrestrial ranges for both δ^13^C and δ^34^S values. Herbivores show variation in δ^34^S values when grouped by region, explained by differences in the local biotopes. This study is the first attempt to connect the Eastern Baltic isotopic baselines and provides more detailed temporal and geographical references to study the local ecologies and interpret the human data.

## Introduction

The adoption of stable isotope analysis to study archaeological questions has revolutionized the field by providing new insight into topics as varied as diet, social equality, palaeoecology, and past movements of people and animals. It has become a staple of multidisciplinary investigations, however, some regions such as the eastern part of Europe have been trailing behind in regards to fully embracing the method, as the arrival of modern scientific methods have reached the area with a certain delay. During recent years, there has been a sizeable increase in the number of publications of stable isotope analyses from the Baltic country of Estonia, yet most data have focused on analyzing human skeletal remains [1–5] with little published evidence of related zooarchaeological material [6].

Proper baseline information is required to accurately reconstruct the nuances of prehistoric and historic dietary habits in the region. The authors of this study have highlighted this need in various papers about the dietary habits of hunter-gatherers [4], Bronze Age individuals [1], and most recently in a study about the δ^13^C, δ^15^N and δ^34^S values from two historic period burial sites in northern Estonia [5]. The latter case-study is particularly valuable, as it enables comparison with written sources that have attested to the great importance of fish in the Medieval menu for both the lower and upper classes [7,8]. However, human stable isotope results have reflected a diet dominated by terrestrial protein with only minor aquatic components [5].

This trend of terrestrial carbon signals coupled with high nitrogen isotope values has also been observed in other Medieval populations from Northern Europe [9–13] and is typically explained by the consumption of a mixed diet including freshwater resources. Yet this contradicts with written accounts which describe the great popularity and availability of (marine) fish, highlighting the need to consider alternative hypotheses. The prevailingly brackish conditions of the Baltic Sea have been shown to obscure the presence of a ‘typical’ marine carbon isotope signal [14–16]. The issue of the potential inclusion of locally caught fish in the diets of Medieval individuals needs further investigation as there is no published stable isotope data for local fish to fully support or reject this hypothesis.

The Medieval case-study from northern Estonia [5] also demonstrated that human δ^34^S values from two historic period cemeteries displayed a surprisingly wide variation, indicating either very diverse food sources or migration between areas with different sulfur isotope baseline values. While the application of sulfur isotope analysis has shown great potential [14,17–24], the interpretation of the results is not always straightforward and is dependent on the existing measurable differences in baseline δ^34^S values between different ecosystems (e.g., marine, terrestrial, freshwater) and/or regions. Having a good overview of the local faunal baseline is therefore crucial for the successful utilization of sulfur stable isotope analysis. It is also necessary to differentiate sulfur isotope variation caused by dietary differences (marine vs terrestrial vs freshwater) from that caused by differences in origin.

This study incorporates the largest and most comprehensive set of faunal bone collagen stable isotope data from prehistoric and historic Estonia. The dataset covers the whole geographical area of present-day Estonia, including 197 faunal skeletal samples (ca. 700–1800 CE) collected during a multidisciplinary project on the foodways of Medieval Estonia (Estonian Research Council grant No PRG29), and 54 samples from the Georeferenced dataset of stable carbon and nitrogen isotope values of prehistoric Estonia and its neighboring areas (ca. 200–1650 CE). By characterizing temporal and geographical trends in isotopic baselines in the area of present-day Estonia we aim to provide an interpretative framework for human paleodietary reconstructions. We will identify the respective local isotope ranges of terrestrial *vs* aquatic, and domesticated *vs* wild species in the later part of Iron Age (200–1225 CE), Medieval (1225–1560 CE) and Early Modern (1560–1800 CE) periods. While this approach is limited in directly following temporal continuity, the main goal is to obtain a regional overview, thus observing temporal or site-specific patterns is of secondary importance. Our research ties into the wider discussion on the need for isotopic baseline datasets for the eastern Baltic Sea region [25].

## Background

### Local environmental and geological conditions

**Fig 1:**
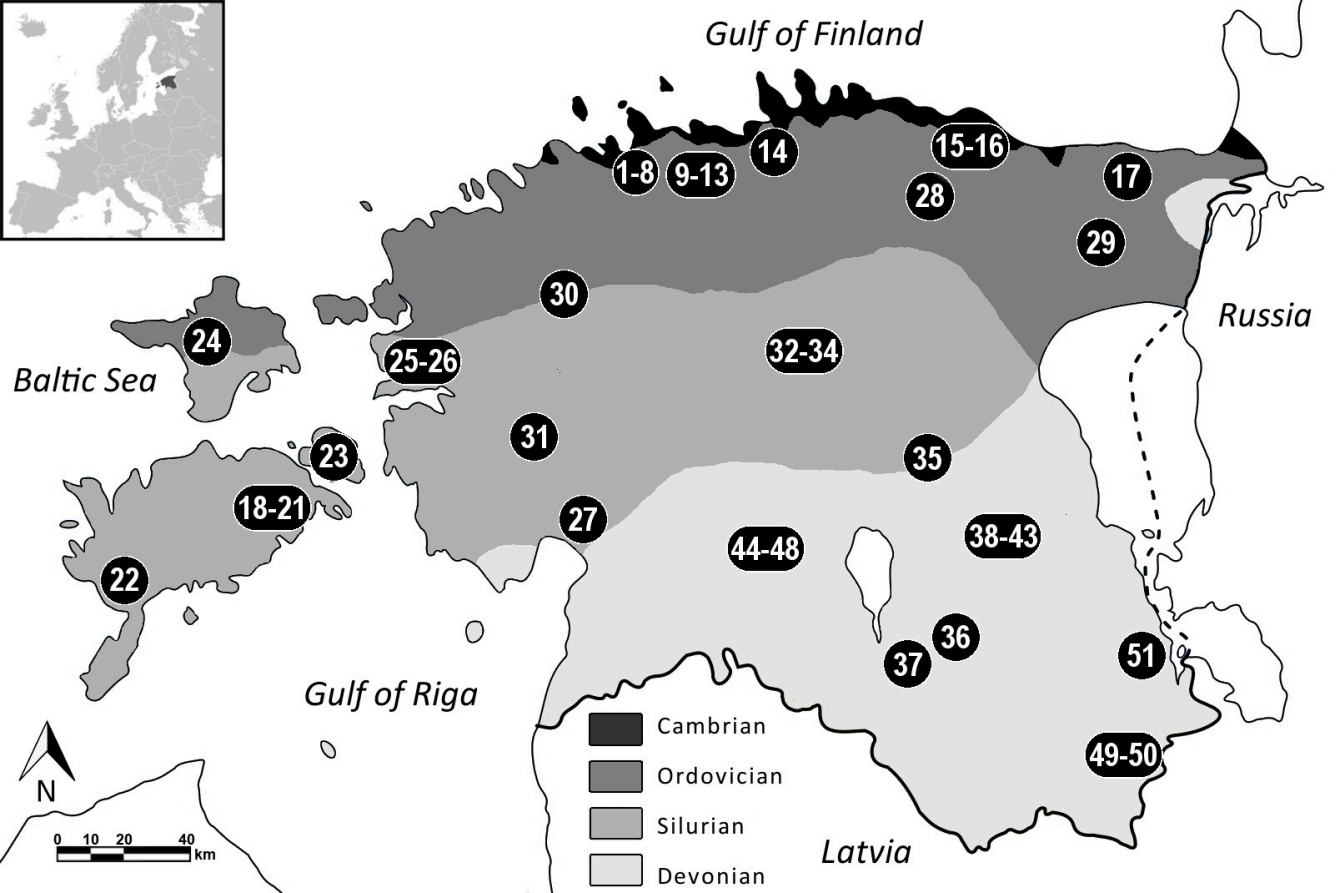
Map of Estonia with bedrock data and location of archaeological sites included in the study. Sites are further divided into ‘northern coastal’ (no. 1–17), ‘western coastal’ (no. 18–27), ‘central inland’ (no. 28–35) and ‘southern inland’ (no. 36–51). Additional site information available in Table 1 and S1 Appendix. Bedrock data drawn after Suuroja [29].

As the northernmost of the three Baltic States, today Estonia enjoys a humid, continental climate with clearly defined seasons and ample forests, rivers and lakes [26]. Surrounded by the Baltic Sea from the north and the west, and the large lake Peipus from the east, it is also characterized by more than 2000 islands (Fig 1). The vegetation is dominated by temperate plants, with typical tree species being pine, spruce, birch and other trees of mixed forests [27]. Common wild mammals include the lynx, grey wolf, brown bear, red fox, badger, wild boar, moose, roe deer, beaver, otter and grey seal [28].

**Table 1:** List of archaeological sites included in the study along with an overview of sampled faunal material. Site numbers (No) correspond to locations on map in Fig 1. Periods are defined as Iron Age (here limited to the later part of the period of ca. 200–1225 CE), Medieval Period (ca. 1225–1560 CE), Early Modern Period (ca. 1560–1800 CE).

No	Archaeological site	Mammals	Birds	Fish	Location	Archaeological context	Notes on context	Period
1	Tallinn, Sauna St	8	0	0	Northern coastal	Urban	Town	Medieval
2	Tallinn, Vabaduse Square	11	0	0	Northern coastal	Urban	Town	Medieval/Early Modern
3	Tallinn, Jahu St	12	0	0	Northern coastal	Urban	Landfill site	Medieval
4	Tallinn, Town Hall Square	4	0	3	Northern coastal	Urban	Town	Medieval
5	Tallinn, Tartu Rd	0	2	4	Northern coastal	Urban	Town	Medieval
6	Tallinn, Tatari St	0	1	0	Northern coastal	Urban	Town	Medieval
7	Tallinn, Kohtu St	0	1	0	Northern coastal	Urban	Upper Town (Elite)	Medieval
8	Tallinn, Härjapea	2	0	0	Northern coastal	Urban	Suburb	Medieval
9	Iru	0	0	2	Northern coastal	Rural	Hill fort	Iron Age
10	Lehmja	11	0	0	Northern coastal	Rural	Settlement site	Iron Age/Medieval/Early Modern
11	Rebala	6	0	0	Northern coastal	Rural	Settlement site	Iron Age/Medieval
12	Rebala	2	0	0	Northern coastal	Rural	Cemetery	Iron Age
13	Jõelähtme	3	0	0	Northern coastal	Rural	Cemetery	Iron Age
14	Muuksi	2	0	0	Northern coastal	Rural	Cemetery	Iron Age
15	Kunda	1	0	0	Northern coastal	Rural	Cemetery	Iron Age
16	Pada	4	0	0	Northern coastal	Rural	Settlement site	Iron Age
17	Kukruse	3	0	1	Northern coastal	Rural	Cemetery	Iron Age/Medieval
18	Valjala	5	0	0	Western coastal	Rural	Hill fort	Iron Age
19	Pöide	6	0	0	Western coastal	Rural	Hill fort	Iron Age
20	Tornimäe	0	0	5	Western coastal	Rural	Harbour site	Iron Age
21	Asva	2	0	0	Western coastal	Rural	Settlement site	Iron Age
22	Salme	1	0	0	Western coastal	Rural	Mixed cultural layer	Early Modern
23	Viira	2	0	0	Western coastal	Rural	Cemetery	Early Modern
24	Kõpu XIV	2	0	0	Western coastal	Rural	Settlement site	Early Modern
25	Haapsalu Castle	0	11	5	Western coastal	Urban	Castle	Early Modern
26	Haapsalu, Jaani St	1	0	0	Western coastal	Urban	Town	Medieval
27	Pärnu, Põhja Blvd	11	1	0	Western coastal	Urban	Town	Medieval/Early Modern
28	Rakvere, Pikk St	9	0	0	Central inland	Urban	Town	Medieval/Early Modern
29	Jõuga	1	0	0	Central inland	Rural	Cemetery	Iron Age
30	Varbola	2	0	0	Central inland	Rural	Hill fort	Iron Age
31	Soontagana	3	1	0	Central inland	Rural	Hill fort	Iron Age
32	Sargvere	12	0	0	Central inland	Rural	Settlement site	Medieval
33	Nurmsi	2	0	0	Central inland	Rural	Cemetery	Iron Age/Early Modern
34	Tarbja	1	0	0	Central inland	Rural	Cemetery	Iron Age
35	Kunila	2	0	0	Central inland	Rural	Cemetery	Early Modern
36	Otepää	11	6	4	Southern inland	Rural	Hill fort	Iron Age/Medieval
37	Kuigatsi	1	0	0	Southern inland	Rural	Hill fort	Iron Age
38	Tartu, Jakobi St	5	1	0	Southern inland	Urban	Town	Medieval
39	Tartu, Lutsu St	6	1	0	Southern inland	Urban	Town	Medieval
40	Tartu, Küütri St	0	0	4	Southern inland	Urban	Town	Medieval
41	Tartu, Dome Church	1	0	0	Southern inland	Urban	Churchyard	Medieval
42	Tartu, University of Tartu Botanical Garden	1	0	0	Southern inland	Urban	Town	Medieval
43	Tartu, Ülikooli St	1	0	0	Southern inland	Urban	Town	Medieval
44	Viljandi Castle Park	1	0	0	Southern inland	Rural	Settlement site	Iron Age
45	Viljandi, Pikk St	6	2	0	Southern inland	Urban	Town	Medieval
46	Viljandi, Viljandi Museum yard	5	1	0	Southern inland	Urban	Town	Medieval
47	Viljandi, Vaksali St	1	0	0	Southern inland	Urban	Town	Medieval
48	Viljandi Castle	10	4	4	Southern inland	Urban	Castle	Medieval
49	Vastseliina Castle	5	0	0	Southern inland	Urban	Castle	Medieval/Early Modern
50	Hinniala	1	0	0	Southern inland	Rural	Hill fort	Iron Age
51	Laossina	1	0	0	Southern inland	Rural	Cemetery	Iron Age
	**Total:**	**187**	**32**	**32**	**251**			

The Baltic Sea, one of the largest brackish-water inland seas in the world, has a diverse hydrological history and has fluctuated between freshwater and marine conditions since the onset of the glacial retreat at the end of Pleistocene. In its present Limnea Sea stage (from ca. 4500 cal. BP) it is characterized by minor inflow of Atlantic saltwater through the narrow Danish Straits and abundant freshwater runoff from the large catchment area of the Baltic Sea [30,31]. The salinity varies widely, both on a horizontal and vertical gradient. It is highest around the Kattegat (ca. 10‰ in surface waters and up to 30‰ in deeper basins) where marine conditions dominate, and reaches its lowest point in the Gulf of Finland and Bothnian Bay (the furthest points from the Danish Straits) where conditions resemble freshwater environments (close to 2–4‰) [31,32].

Estonia has a moderately varied topography with the main features being an extensive limestone plateau in the northern part of the country, a relatively flat low-lying area in the west and islands, and gently sloping hills in the south [33]. The pre-Quaternary bedrock comprises Ordovician and Silurian limestones and dolomites in the north and Devonian sandstone in the south, with a narrow band of Cambrian clay and sandstone outcrop on the northern coastline (see also Fig 1) [31,34,35]. The landscape has been shaped by glacial processes such as erosion (in the north and west) and accumulation (in the south) of Pleistocene deposits, in addition to glacio-isostatic land uplift, which has resulted in extensive shore displacement throughout the Holocene [31,35].

### Stable isotope biogeochemistry

The basic principles of stable isotope analysis and its use in archaeology are well established [36,37]. It is based on the premise that bone collagen reflects the isotopic composition of foods (predominantly the protein portion of the diet) consumed over several years before death [38–42].

Carbon isotope ratios (δ^13^C) are used to distinguish between marine and terrestrial sources of carbon, but also between diets based on either C_3_ or C_4_ plants [36,43]. There is a trophic level enrichment in δ^13^C of about 5‰ between plants and herbivorous animals, and about 1‰ between carnivores and their prey [37,38,44]. Freshwater food webs exhibit widely varying C-isotope signatures, which are generally more negative than marine species but can range in their δ^13^C anywhere between -12‰ and -28‰ [11,45,46].

Nitrogen isotope ratios (δ^15^N) broadly reflect the trophic level of the individual, as δ^15^N values increase by about 3–6‰ with every step up the food chain [36,43,47,48]. Because of the greater number of steps in the marine food chain, fish and marine mammals typically exhibit the highest δ^15^N values. Several factors can influence the nitrogen isotopic composition of plants and animals, e.g., climatic conditions such as precipitation and temperature [49,50], the usage of nitrogen-based fertilizers [51–53] and the ‘nursing effect’ observed in suckling animals [54–57].

Sulfur isotope ratios (δ^34^S) in terrestrial plants have been shown to vary significantly due to different sulfur sources in the local geology, with additional sulfate sources from the hydrosphere (e.g., ground and stream water) and atmosphere (e.g., sulfur dioxide SO_2_) all contributing to the average δ^34^S values of the local biologically available sulfur [21,58–61]. Freshwater environments can display an equally wide range in their δ^34^S values so that there is a lot of potential overlap between the terrestrial and freshwater ecosystems [18,20,21,59]. In contrast, δ^34^S values of marine organisms living in the ocean are fairly constant and fall within a relatively narrow range between 17‰ and 21‰ [59,62]. Coastal regions can also be affected by the ‘sea-spray effect’, where the presence of marine sulfates increases soil δ^34^S values, resulting in plants grown on these soils having sulfur isotope values similar to the marine ecosystem [61,63]. This effect decreases with increasing distance from the coast, extending from a few kilometers inland to covering whole islands [63,64].

## Material and methodology

### Overview of sites and samples

A wide selection of sites was included to create a comprehensive isotopic baseline of the whole study area (Fig 1 and Table 1). All distinct regions of Estonia are represented, divided into four sub-regions based on geographical and geological features. ‘Northern coastal’ and ‘western coastal’ include sites situated up to 10 km from the (modern) coastline. ‘Western coastal’ also encompasses the largest islands of Saaremaa, Hiiumaa and Muhu. Inland regions are further divided into ‘central inland’ and ‘southern inland’ with the latter roughly corresponding to the geologically distinct area dominated by Devonian sandstone (see Fig 1).

**Fig 2:**
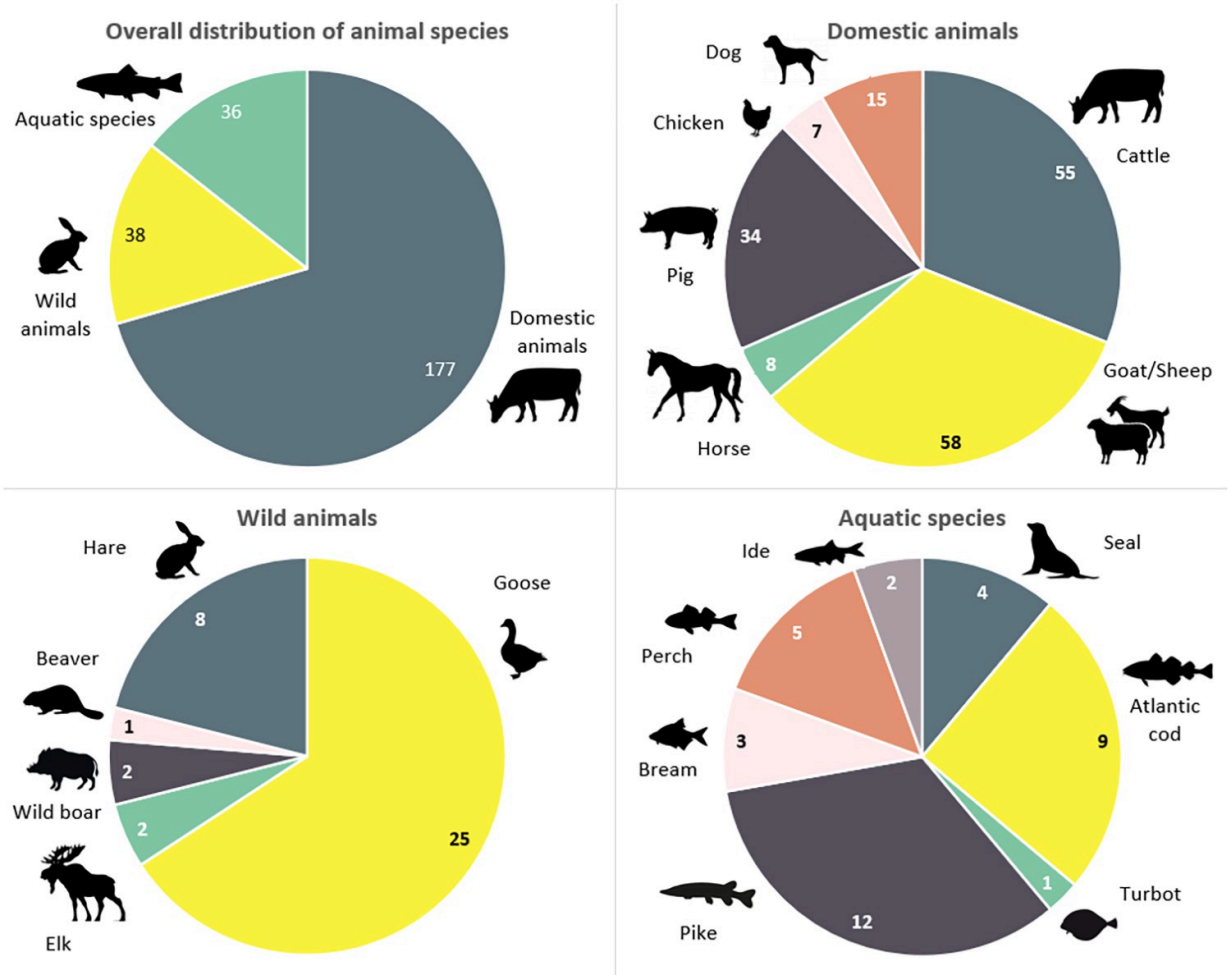
Distribution of terrestrial, avian and aquatic species sampled for this study. Cattle (*Bos taurus*), sheep (*Ovis aries*), goat (*Capra hircus*), horse (*Equus caballus*), pig (*Sus domesticus*), chicken (*Gallus gallus domesticus*), dog (*Canis familiaris*), goose (*Anser* sp./*Branta* sp.), elk (*Alces alces*), wild boar (*Sus scrofa*), beaver (*Castor fiber*), hare (*Lepus* sp.), seals (*Phocidae*), Atlantic cod (*Gadus morhua*), turbot (*Scophthalmus maximus*), pike (*Esox lucius*), bream (*Abramis brama*), perch (*Perca fluviatilis*), ide (*Leuciscus idus*).

Individual sample selection was based on availability of identified faunal remains in the chosen zooarchaeological assemblages. Altogether 251 samples representing the main sources of animal protein available to the local people were collected, including the most common domestic and wild terrestrial and avian species, and various marine and freshwater fish (Fig 2). Although geese (*Anser* sp./*Branta* sp.) specimens are listed under ‘wild animals’, it is possible that some of these were likely domestic birds [65].

Temporal parameters of each sample are assigned based on archaeological context and, where possible, radiocarbon dating (for 67 out of 251 samples, see S1 Appendix for calibrated dates). To facilitate data analysis, three distinct temporal categories are used (see Table 1), which reflect potential historical differences in settlement pattern, land management, and resource use and availability [66,67]. A best-fit policy was adopted to deal with such a diverse dataset (e.g., when a sample was dated to a transitional period, or the archaeological context spanned a wide temporal range). We recognize that this may obscure the detection of actual temporal patterns, however, this subject can be addressed in future studies.

Based on archaeological context, all sites were further categorized as either ‘rural’ or ‘urban’. ‘Rural’ sites include Medieval and Post-Medieval villages but also all Iron Age sites (e.g., hill forts, settlement and burial sites). The rural societies from the Iron Age until the Early Modern Period were small agrarian villages by their structure that formed larger parishes [67]. In essence, ‘rural’ sites can be tentatively considered to represent locally raised and caught animals. ‘Urban’ sites are exclusively from the Medieval and Early Modern periods (including Medieval castles and Hanseatic towns), characterized by higher population density, religious (i.e. Christian) influences, and–in addition to the resources brought from their hinterlands–access to more diverse (food) resources, including imported fish.

### Methodology and quality indicators

All necessary permits were obtained for the described study, which complied with all relevant regulations. Sampling permission was granted by the Archaeological Research Collection at Tallinn University, Estonia (sampling protocols AI PP no. 497, 498, 501, 502, 505, 506, 515, 529–531, 600, 604, 605, 612, 613, 617, 618, 620, 621, 623, 624, 630, 631), Institute of History and Archaeology at the University of Tartu, Estonia (sampling protocols TÜ PP no. 102, 120–130) and Tartu City Museum, Estonia (sampling protocols TM PP no. 2, 4). Sampling protocols (with photographs before and after sampling) are stored with the respective collection holders. Specimen numbers and other contextual information can be found in S1 Appendix.

The stable isotope and radiocarbon data presented in this paper have been assembled from analytical work conducted in several establishments. Of the total of 251 samples, 197 were processed for collagen extraction in the Biochemistry Laboratory of the School of Natural Sciences and Health at Tallinn University, Estonia. Stable δ^13^C, δ^15^N and δ^34^S isotope analysis for these samples was conducted at the Scottish Universities Environmental Research Centre (SUERC) Radiocarbon Laboratory in East Kilbride, United Kingdom. Radiocarbon ages were obtained from selected samples (n = 13) at the Poznań Radiocarbon Laboratory in Poland. For the remaining 54 samples, collagen extraction and radiocarbon dating was performed at the Leibniz Laboratory for Radiometric Dating and Stable Isotope Research, at the Christian-Albrechts-University of Kiel, and stable isotope measurements done at the Isolab GmbH, Germany. Thirteen (n = 13) samples were further analyzed with Zooarchaeology by Mass Spectrometry (ZooMS) in the BioArCh lab at the University of York, United Kingdom, to differentiate between goat and sheep (S2 Appendix). A full methodological description along with details on the analytical procedure and equipment used is presented in S3 Appendix.

To check sample quality, routinely used criteria were followed [68]. A few samples (n = 8) did not produce enough collagen for δ^13^C, δ^15^N and δ^34^S analysis. Most samples had atomic C:N ratios within the generally acceptable range of 2.9 to 3.6 [68,69], except for two specimens. One of them (cattle–*GUsi10837*) had a C:N ratio of 3.7 but satisfactory elemental concentrations, so was included in the dataset. The other (hare–*GUsi10812*) had a ratio of 3.8 but since this sample also had problematic δ^34^S quality indicators, it was discarded from further analysis. For carbon and nitrogen concentrations, most samples were above 30% for %C and more than 10% for %N, indicative of good quality collagen [68]. Seven samples had both values below that range but still within the accepted lower limits of 13% for %C and 5% for %N [70].

Quality indicators for sulfur stable isotopes are not as established as for δ^13^C and δ^15^N. Arguably, the research concerning these criteria is still in progress as the number of controlled, large-scale studies on δ^34^S isotopes is relatively low. In our study, mammalian and avian samples have an average C:S atomic ratio of 482 ± 96 (1-sigma standard deviation), N:S atomic ratio of 144 ± 29 (1SD), and sulfur concentration (%S) of 0.24% ± 0.1% (1SD), consistent with results reported by other researchers [14,17,23,71]. We have excluded nine samples with C:S/N:S ratios of <250/75 and %S >0.40% as these values may represent sulfur contamination. The δ^13^C and δ^15^N values for these samples were not excluded as the other collagen quality indicators were acceptable.

Fish atomic C:S and N:S ratios reported here (on average 254 ± 34 and 81 ± 12, respectively) are somewhat higher than those seen in other studies [18,23,71] and the %S lower (0.42% ± 0.1%). However, all fish samples have acceptable %C and %N content, C:N ratios and visual appearance. There has not been enough research done on fish δ^34^S to argue whether the reported values may be influenced by contamination. Since it seemed to us that these data were still meaningful, we chose to include them in our study.

Statistical analysis was applied to determine whether differences between groups of data were (statistically) significant. A non-parametric Mann–Whitney U test was preferred due to the presence of outliers and non-normally distributed data (as assessed by the Shapiro–Wilk test of normality). When more than two groups were compared, the non-parametric Kruskal–Wallis H test was applied. The rejection of the null hypothesis for the Kruskal–Wallis test was automatically followed by a post hoc analysis using Dunn’s procedure with a Bonferroni correction for multiple comparisons. Statistical uncertainty was set at 95%.

## Results and discussion

**Fig 3:**
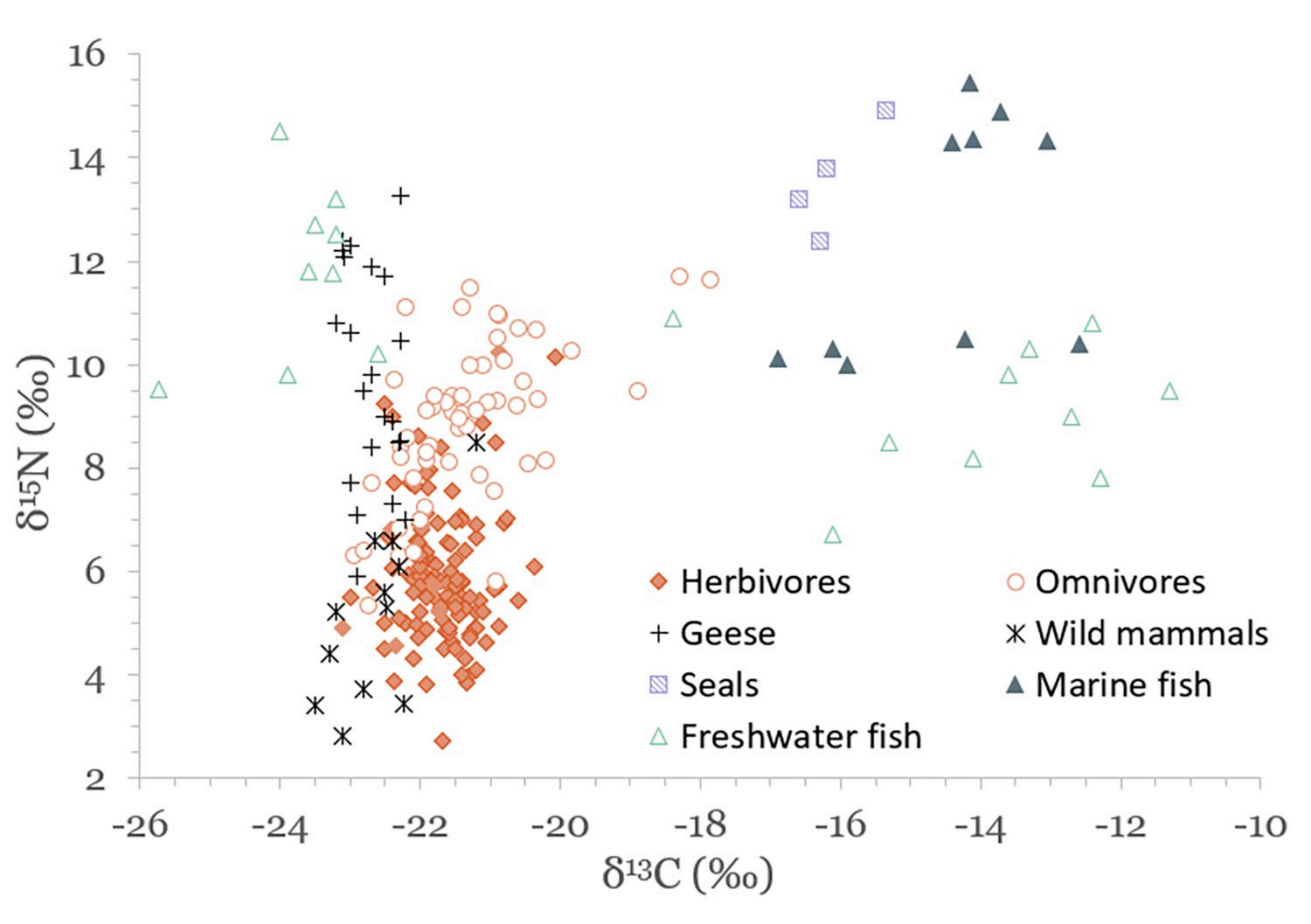
Scatterplot of stable carbon and nitrogen isotope data for faunal specimens.

**Fig 4:**
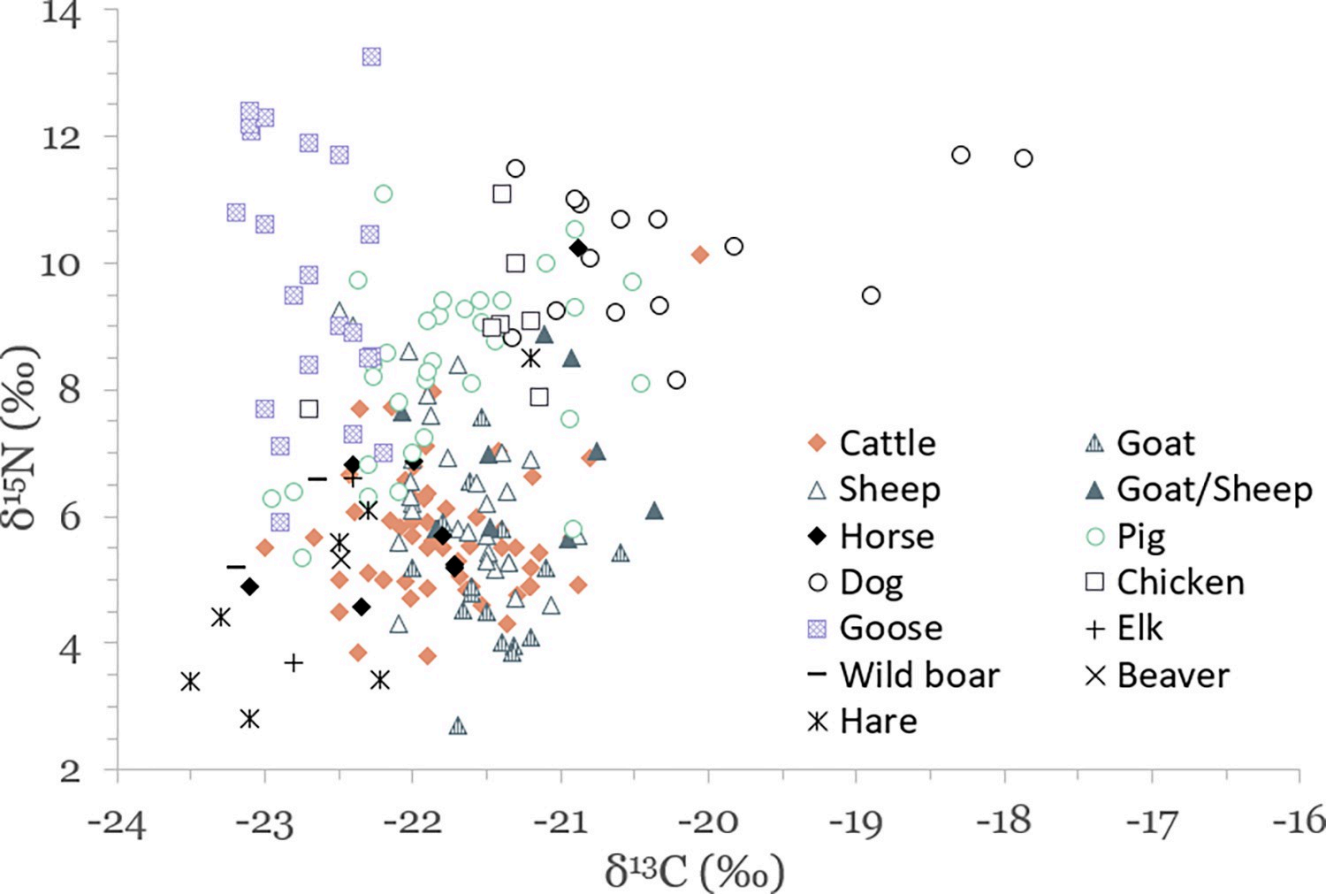
Scatterplot of stable carbon and nitrogen isotope data for terrestrial species.

**Fig 5:**
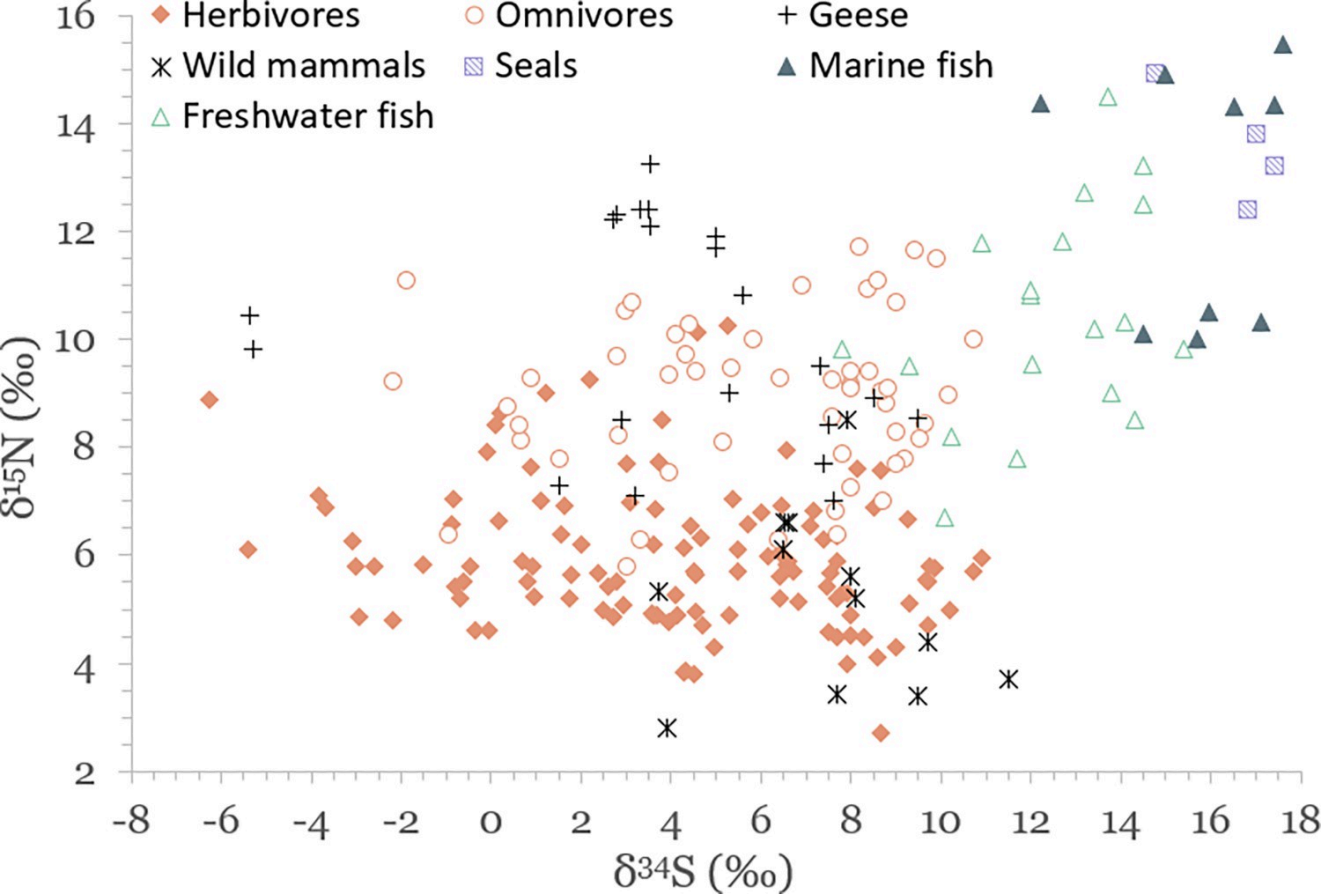
Scatterplot of stable sulfur and nitrogen isotope data for faunal specimens. Aquatic species included for reference.

Results of stable isotope analyses are summarized in Table 2 and plotted in Figs 3–5. A full list of analytical measurements along with associated radiocarbon ages, contextual and collagen quality information can be found in S1 Appendix. Additional summary statistics and results of statistical tests of significance are presented in S4 Appendix.

**Table 2:** Summary statistics of stable carbon, nitrogen and sulfur isotope data of analyzed faunal samples by group (number of samples that produced results, average, 1-sigma standard deviation, minimum and maximum values, range). Goose δ^13^C and δ^15^N results have been previously published [60].

Group (n)		δ^1^^3^C (‰)					δ^1^⁵N (‰)					δ^3^⁴S (‰)				
		AVG	SD	Min	Max	Range	AVG	SD	Min	Max	Range	AVG	SD	Min	Max	Range
Cattle (54)		-21.8	0.5	-23.0	-20.1	2.9	5.7	1.1	3.8	10.1	6.3	4.1	3.9	-3.8	10.9	14.7
Caprines total (56)		-21.6	0.4	-22.5	-20.4	2.1	6.1	1.4	2.7	9.3	6.6	4.1	4.1	-6.3	9.9	16.2
	Goat (17)	-21.5	0.3	-22.0	-20.6	1.4	5.0	1.2	2.7	7.6	4.9	5.4	4.0	-2.6	8.7	11.3
	Sheep (30)	-21.7	0.4	-22.5	-20.9	1.6	6.4	1.3	4.3	9.3	5.0	3.8	4.0	-5.4	9.9	15.3
	Goat/Sheep (9)	-21.2	0.5	-22.1	-20.4	1.7	6.9	1.2	5.7	8.9	3.2	2.8	4.6	-6.3	9.8	16.1
Horse (8)		-22.0	0.6	-23.1	-20.9	2.2	6.2	1.9	4.6	10.3	5.7	4.5	2.4	1.0	7.5	6.5
**Domestic herbivores (118)**		**-21.7**	**0.5**	**-23.1**	**-20.1**	**3.0**	**5.9**	**1.3**	**2.7**	**10.3**	**7.6**	**4.1**	**3.9**	**-6.3**	**10.9**	**17.2**
Pig (34)		-21.8	0.6	-23.0	-20.5	2.5	8.3	1.4	5.3	11.1	5.8	4.9	3.4	-1.9	9.6	11.5
Dog (15)		-20.2	1.1	-21.3	-17.9	3.4	10.2	1.1	8.2	11.7	3.5	6.4	3.3	-2.2	9.9	12.1
Chicken (7)		-21.5	0.5	-22.7	-21.1	1.6	9.1	1.2	7.7	11.1	3.4	9.1	1.0	7.8	10.7	2.9
**Domestic omnivores (56)**		**-21.3**	**1.0**	**-23.0**	**-17.9**	**5.1**	**8.9**	**1.6**	**5.3**	**11.7**	**6.4**	**5.9**	**3.4**	**-2.2**	**10.7**	**12.9**
Goose (23)		-22.7	0.3	-23.2	-22.2	1.0	9.9	2.1	5.9	13.3	7.4	4.1	3.8	-5.4	9.5	14.9
Elk (2)		-22.6	0.3	-22.8	-22.4	0.4	5.2	2.1	3.7	6.6	2.9	9.1	3.5	6.6	11.5	4.9
Wild boar (2)		-23.0	0.4	-23.2	-22.7	0.5	5.9	1.0	5.2	6.6	1.4	7.3	1.1	6.5	8.1	1.6
Beaver (1)		-22.5	N/A	N/A	N/A	N/A	5.3	N/A	N/A	N/A	N/A	3.7	N/A	N/A	N/A	N/A
Hare (7)		-22.6	0.8	-23.5	-21.2	2.3	4.9	2.0	2.8	8.5	5.7	7.6	2.0	3.9	9.7	5.8
**Wild mammals (12)**		**-22.6**	**0.6**	**-23.5**	**-21.2**	**2.3**	**5.1**	**1.7**	**2.8**	**8.5**	**5.7**	**7.5**	**2.3**	**3.7**	**11.5**	**7.8**
Seal (4)		-16.1	0.5	-16.6	-15.4	1.2	13.6	1.1	12.4	14.9	2.5	16.5	1.2	14.8	17.4	2.6
Cod (9)		-14.7	1.3	-16.9	-13.1	3.8	12.7	2.4	10.0	15.5	5.5	15.8	1.7	12.2	17.6	5.4
Turbot (1)		-12.6	N/A	N/A	N/A	N/A	10.4	N/A	N/A	N/A	N/A	14.8	N/A	N/A	N/A	N/A
**Marine fish (10)**		**-14.5**	**1.4**	**-16.9**	**-12.6**	**4.3**	**12.5**	**2.4**	**10.0**	**15.5**	**5.5**	**15.7**	**1.6**	**12.2**	**17.6**	**5.4**
Pike (10)		-19.6	5.3	-24.0	-11.3	12.7	11.4	1.6	9.5	14.5	5.0	12.1	2.2	7.8	14.5	6.7
Perch (4)		-18.3	5.9	-23.6	-12.7	10.9	10.8	1.7	9.0	12.5	3.5	14.1	1.1	12.7	15.4	2.7
Bream (3)		-19.0	5.8	-25.7	-15.3	10.4	8.2	1.4	6.7	9.5	2.8	12.1	2.1	10.1	14.3	4.2
Ide (2)		-13.2	1.3	-14.1	-12.3	1.8	8.0	0.3	7.8	8.2	0.4	11.0	1.1	10.2	11.7	1.5
**Freshwater fish (19)**		**-18.6**	**5.2**	**-25.7**	**-11.3**	**14.4**	**10.4**	**2.0**	**6.7**	**14.5**	**7.8**	**12.4**	**2.0**	**7.8**	**15.4**	**7.6**

Due to the diverse dataset, it was not possible to follow a rigid sampling procedure, e.g., only sampling specific skeletal element(s) or only adult (mature) animals. We acknowledge that these factors may result in data that are not entirely comparable due to minor isotopic differences caused by intraskeletal and interspecies variation in bone collagen turnover rates [72–75] and age-specific diets [54–57]. However, the discrepancies in laboratory protocol and analytical equipment between the two sets of analyzed data should not hinder their meaningful comparison as has been demonstrated by various inter-laboratory experiments [76,77]. It is unlikely that the listed differences are significant enough to obscure any major patterns that may exist in the local baseline.

### Carbon and nitrogen isotopic variation in the terrestrial food web

Domestic herbivores have average values of –21.7 ± 0.5‰ (1SD) for δ^13^C and 5.9 ± 1.3‰ for δ^15^N, domestic omnivores –21.3 ± 1.0‰ and 8.9 ± 1.6‰, wild mammals –22.6 ± 0.6‰ and 5.1 ± 1.7‰, and geese –22.7 ± 0.3‰ and 9.9 ± 2.1‰ (Table 2 and Fig 3). These results reflect a temperate, C_3_-plant dominated ecosystem, characteristic to local flora. Domestic herbivores and omnivores have statistically significantly different δ^13^C and δ^15^N values (Mann–Whitney U test, p<0.03 for both variables) due to the inclusion of animal protein in the diet of omnivorous animals.

#### Domestic herbivores

Among domestic herbivores, there is a particularly large variation in both δ^13^C and δ^15^N values. δ^13^C values range from –20.1‰ to –23.1‰. There are no statistical differences in δ^13^C values between domestic herbivores found from urban *vs*. rural contexts (S4 Appendix). Lower δ^13^C values (below –22‰) were likely due to forested environments [78,79], e.g., animals left to graze freely in the surrounding woodlands, whereas higher δ^13^C values can be associated with animals raised in open areas, probably close to human settlements. However, there are no statistically significant differences between domestic herbivore δ^13^C values by region, nor when only comparing coastal *vs*. inland samples. Although terrestrial plants display geographical variation in stable carbon isotope ratios due to climatic conditions such as water availability, temperature, and sunlight exposure [40,80,81], these factors probably influence herbivore isotope values on a much larger scale than observed here. Herbivore carbon isotopic values also show no statistical differences between periods, suggesting relative consistency in herding conditions from the later Iron Age to Medieval and Post-Medieval times.

Domestic herbivore δ^15^N values range from 2.7‰ to 10.3‰. The large variation can be explained by various scenarios which are not necessarily mutually exclusive. For example, atypically high δ^15^N values in herbivores can be caused by nursing, i.e. suckling animals literally feeding off their mothers [54–57]. Regrettably, it is not always possible to determine the exact age of the animal (i.e. whether it was a suckling) based on a single bone or bone fragment. Of the two herbivores that can be considered as outliers based on their high δ^15^N (and δ^13^C) values (δ^15^N 10.1‰ and δ^13^C –20.1‰–cattle, *KIA-55267*; and δ^15^N 10.3‰ and δ^13^C –20.9‰–horse, *KIA-55263*; see S1 Fig), the former was an adult individual and the latter a juvenile. This implies that while increased δ^15^N values *may* be due to the inclusion of young animals, it was certainly not the only factor influencing the observed variation.

The usage of traditional fertilizers such as animal manure on plants can also increase their δ^15^N values by a full trophic level [51–53]. If this enrichment is carried up the food chain, it can lead to herbivores having δ^15^N values characteristic to omni- or carnivores. Low δ^15^N values could thus reflect consumption of wild plants, whereas higher values can be due to animals grazing on cultivated land or being directly fed manured crops. Statistical analyses demonstrated that urban domestic herbivore δ^15^N values were significantly different from rural herbivore δ^15^N values (Mann–Whitney U test, U = 1468.5, p = 0.007) with the latter having on average higher δ^15^N values (Table 3). This would imply that rural herbivores were consuming more intensively manured plants or that they were more often slaughtered at young age compared to urban herbivores. However, the distinction between urban and rural domestic herbivores may be altogether misleading as even in the largest towns, herbivores usually grazed outside the town limits, and livestock may have been brought to towns for butchering or selling at the market from the surrounding rural areas.

**Table 3:** Comparison of mean and standard deviations (1-sigma) of domestic herbivore δ^13^C and δ^15^N values based on site, geographical region and temporal group.

Context	n	δ^13^C (‰)	δ^15^N (‰)
Urban	60	–21.8 ± 0.5	5.6 ± 1.3
Rural	58	–21.6 ± 0.6	6.2 ± 1.3
Northern coastal	48	–21.7 ± 0.5	5.9 ± 1.4
Western coastal	21	–21.6 ± 0.6	5.9 ± 1.1
Central inland	19	–21.6 ± 0.6	6.1 ± 1.2
Southern inland	30	–21.8 ± 0.6	5.8 ± 1.4
Iron Age	31	–21.5 ± 0.6	6.2 ± 1.5
Medieval	73	–21.7 ± 0.4	5.7 ± 1.2
Early Modern	14	–21.7 ± 0.5	6.4 ± 1.3
**All**	**118**	**–21.7 ± 0.5**	**5.9 ± 1.3**

In our dataset the majority of ‘rural’ samples are from Iron Age contexts. Iron Age domestic herbivores do have on average higher δ^15^N (and δ^13^C) values compared to Medieval ones, although the differences between chronological contexts are not statistically significant (Kruskal–Wallis H test, H = 4.419, d.f. = 2, p = 0.110 for δ^15^N; but H = 3.436, d.f. = 2, p = 0.179 when two outliers with high δ^15^N values are removed). We are not sure why Medieval herbivores would have had lower δ^15^N values compared to Iron Age and Early Modern animals (Table 3). If agricultural activities and manuring practices intensified through time, we would have expected Iron Age herbivores to display the lowest δ^15^N values, not Medieval. But animal husbandry practices may have differed between these periods.

A general trend in global soil δ^15^N values has been recognized, which seems to decrease with increasing annual precipitation and decreasing mean annual temperature [49]. Similarly, European terrestrial herbivore δ^15^N values show a distinct decline during the Last Glacial Maximum compared to earlier and later phases when the climate was milder [50]. The only significant climatic fluctuation during the study period was the Little Ice Age [82,83], which could have theoretically resulted in decreased herbivore δ^15^N values. However, the LIA peaked in the Early Modern period (ca. 16–19^th^ centuries), having been preceded by relatively warmer conditions in Northern Europe during the so-called Medieval Warm Period (ca. 9–14^th^ centuries) [84]. As such, we would expect this to have produced an opposite δ^15^N trend to what is observed here. It thus seems unlikely that climate had a significant effect on terrestrial herbivore nitrogen isotopic values in our dataset.

Considering that many of the included sites were in continuous use, non-radiocarbon dated samples may have been affected by potential mixing of earlier and later material. However, only comparing radiocarbon dated samples does not significantly change the outcomes regarding average group values and results of statistical tests of significance, nor are the results notably affected when removing the two outliers (S4 Appendix). There are also no consistent differences between Iron Age and Medieval δ^15^N values when analyzed by species (see S2 Fig, S4 Appendix). This taken together implies that there are no systematic differences in domestic herbivore nitrogen isotope values between Iron Age and Medieval periods, and that the underrepresentation of Iron Age (and Early Modern) samples may have skewed the results of statistical comparisons.

Some of the variation in domestic herbivore δ^13^C and δ^15^N values is certainly due to inter-species differences, although statistically only Medieval period goats and sheep had significantly different δ^15^N values (S4 Appendix; all other comparisons between species, either combined or by period, failed to produce significant differences). Additionally, mean values of herbivorous species are not that different from each other, and it is evident from Fig 4 that intra-species isotopic variation is sometimes even greater than inter-species variation, suggesting that species-specific differences were not the main factor behind the observed variation. The wide variation in domestic herbivore δ^13^C and δ^15^N values may have also been due to sampling different sites across a large area. The notable range of herbivore isotopic variation seems to indicate that domesticates were handled in varying conditions depending on available resources and conditions specific to any given site.

#### Domestic omnivores

For domestic omnivores, all three species–the pig, dog and chicken–are distinct in their isotopic distribution pattern. The wide range of isotopic variation for omnivores is to be expected considering they have the option to alternate between herbivorous and carnivorous diets. δ^13^C values range from –17.9‰ to –22.8‰ (4.9‰ total) and δ^15^N values from 5.3‰ to 11.7‰ (6.4‰ total). The range of omnivore δ^15^N values is in fact smaller than was reported for domestic herbivores (7.6‰; although this was due to the inclusion of two outliers with high δ^15^N). There are no statistical differences between urban and rural omnivore δ^13^C and δ^15^N values. Statistical comparisons between omnivores from different regions and periods were run (S4 Appendix), but due to the limited number of samples in some of the groups, the results may be skewed and will thus not be discussed further here.

Pigs show the most variation in δ^15^N values, and dogs in δ^13^C values. Pigs also have the lowest mean δ^13^C and δ^15^N values and dogs the highest, whereas chickens (although with a limited number of samples) tend to plot in-between. There is a positive significant correlation between pig δ^13^C and δ^15^N values (Pearson’s R = 0.43, p = 0.011 for all values, but R = 0.77, p<0.001 when removing four outliers: *GUsi9209*, *KIA-55257*, *GUsi10831*, *GUsi10844*), suggesting that variation in pig isotope values can be best explained by increased reliance on higher trophic level protein. Pigs sampled here range from almost fully herbivorous (possibly left to roam freely in forests) to those similar to chicken and dog. However, most pigs show little overlap with ruminants when compared with samples from the same site (e.g., urban areas such as Tallinn, Tartu, Viljandi and Pärnu where at least ten domesticated animals were analyzed), suggesting that those on predominantly herbivorous diets were not consuming the same plants as (most) cattle and caprids were.

Dogs are the most distinct of all omnivores, indicating a unique diet, and are the only terrestrial species with δ^13^C values above –20‰. Dogs’ isotopic values can sometimes be used as a proxy for human diet due to their role as companions and pets. Indeed, published human isotopic data from two historic period sites in northern Estonia [5] exhibit notable resemblance to dogs’ values, especially for urban contexts. Individuals buried at the suburban St Barbara cemetery (15^th^–18^th^ century) have mean values of –20.0‰ and 11.2‰ for δ^13^C and δ^15^N compared to –20.0‰ and 10.7‰ for urban dogs in this study. For rural dogs, the mean values of –20.5‰ and 9.6‰ for δ^13^C and δ^15^N are more distinct from rural humans buried at the Kaberla village cemetery (12^th^–17^th^ century), who have mean values of –19.8‰ and 10.4‰. The larger discrepancy between rural dogs and humans may be due to the fact that the cemetery is from northern coastal Estonia whereas the majority of rural dogs sampled for this study are from inland sites further south. However, the close similarity of urban commoners’ and dogs’ isotope values from similar contexts supports the assumption that dogs were likely fed household scraps and leftovers, including some aquatic resources as evidenced by two dogs (both from the same urban coastal Medieval context) with δ^13^C values of –17.9‰ and –18.3‰.

#### Wild mammals

Wild mammals have, in general, lower δ^13^C and δ^15^N values compared to most domesticated animals. Statistical comparisons between wild and domestic herbivores demonstrate significant differences in their δ^13^C values (Mann–Whitney U test, U = 149, p<0.0001), but not for nitrogen (U = 383, p = 0.066). No similar comparisons were run for omnivores due to their fewer numbers in the dataset. The isotopic range of wild mammals reported here is characteristic of forested environments and the consumption of wild (i.e. non-cultivated) plants. There is a noticeable range of isotopic variation observed for hares, most of which are from similar contexts (Medieval southern inland), probably reflecting differences in habitat and food availability. One hare is a clear outlier (*GUsi10234*, see also Fig 4) that may have been kept in captivity (e.g., as a pet).

Wild boars have isotope values that partly overlap with some of the domestic pigs, which supports the assumption that pigs were sometimes left to roam freely in forests. While it is often not possible to distinguish between the wild boar and domestic pig in the zooarchaeological material, the animals from the periods under study are quite distinctive in their size, with Iron Age and Medieval pigs being much smaller than their wild ancestors.

#### Geese

Geese comprise a separate group as domestic and wild geese are hard to differentiate based on bone morphometrics alone. While at least some of the geese sampled here were most likely domestic [65], specifically those with high δ^15^N values indicative of fattening, it is still noteworthy that geese occupy a completely unique niche among other sampled terrestrial fauna. Their low δ^13^C values are indicative of influences from freshwater environments and/or uncultivated landscapes.

### Sulfur isotopic variation in the terrestrial food web

Terrestrial animals display a remarkably wide range (17.8‰) in their δ^34^S values, from –6.3‰ to 11.5‰ (average 4.8 ± 3.8‰) (Figs 5 and S3). There is also a great deal of overlap between different species. Terrestrial animals as a group have statistically significantly different δ^34^S values compared to both marine and freshwater species (p<0.0001).

Animals obtain their sulfur through dietary protein (either plant- or animal-based) with only a minor (on average +0.5‰) trophic level change between consumer and food source [44,61,85]. Therefore, animals eating in a specific region should have δ^34^S values similar to the surrounding terrestrial vegetation. Because of this and how the local sulfur isotopic baseline has remained relatively constant in (pre)history, the observed variation in our dataset is unlikely to have been caused by species- or period-specific differences. For this reason, sulfur isotopic data should be analyzed by region.

In Table 4, average terrestrial δ^34^S values from the four regions are compared with each other, demonstrating noticeable and statistically significant (except for between ‘northern coastal’–‘western coastal’ and ‘western coastal’–‘central inland’) differences between them (see S4 Appendix for results of statistical tests). There appears to be a north-west/south-east gradient with δ^34^S values increasing further south, ranging from –6.3‰ in ‘northern coastal’ to 11.5‰ in ‘southern inland’.

**Table 4:** Regional average δ^34^S values of terrestrial fauna (with 1SD), compared with only terrestrial herbivores and only terrestrial omnivores.

Region	All terrestrial (n = 209)	Herbivores (n = 128)	Omnivores (n = 81)
Northern coastal	1.9‰ ± 3.8‰	1.7‰ ± 3.6‰	3.4‰ ± 3.5‰
Western coastal	3.9‰ ± 3.0‰	3.5‰ ± 3.7‰	5.7‰ ± 2.3‰
Central inland	5.0‰ ± 2.8‰	5.1‰ ± 2.5‰	4.8‰ ± 3.5‰
Southern inland	7.8‰ ± 1.7‰	7.8‰ ± 1.7‰	8.3‰ ± 1.3‰

Since the terrestrial fauna also includes omnivorous animals, whose δ^34^S values may have been influenced by direct or indirect consumption of aquatic protein, the regional average values may not display the true terrestrial sulfur signal obtained through local plants. In Table 4, δ^34^S values of all terrestrial species are further compared with only herbivorous animals (both wild and domestic) and only omnivorous animals (including geese). While geese are predominantly herbivorous, their δ^34^S values cannot be considered a reliable indicator of the local terrestrial baseline due to their preference for aquatic plant consumption, in addition to the possibility that some sampled geese may have been wild (i.e. migratory) birds. However, removing the geese samples from analysis altogether does not significantly change the outcome, as most specimens actually plot near the regional mean δ^34^S value, which may suggest that they were locally raised.

The differences between regional average δ^34^S values remain consistent even if only terrestrial herbivores are included in the analysis (Table 4). While the mean values and 1SD are very similar between the ‘all terrestrial’ and ‘herbivore’ groups, again, statistically ‘northern coastal’–‘western coastal’ and ‘western coastal’–‘central inland’ are not significantly different.

Omnivore mean δ^34^S values display a slightly different pattern with coastal regions demonstrating distinct (but not statistically significant) differences between omnivore and herbivore mean δ^34^S values, with the former having on average around 2‰ higher values compared to the latter. This variance is best explained by the consumption of aquatic protein (with higher δ^34^S values) by omnivorous animals, which has resulted in an increase of local terrestrial δ^34^S values. Inland regions do not show these differences. It is unlikely that this is due to inland omnivores not consuming any aquatic protein; the lack of difference is rather caused by the local terrestrial δ^34^S baseline already being higher there.

The increase in average coastal omnivore δ^34^S values may also reflect an inclusion of dietary sources affected by the ‘sea-spray effect’, which results in increased δ^34^S values due to the effects of airborne marine sulfates on coastal soils and plants [61,63]. However, no consistent sea-spray effect was observed among coastal herbivores. Additionally, coastal fauna have on average the lowest δ^34^S values in the whole dataset, although some herbivores from coastal areas do have noticeably high δ^34^S values. The absence of a consistent sea-spray effect can be taken to suggest that either its range is relatively limited and/or that most of the sampled terrestrial fauna were not feeding close to the shoreline. However, without sampling local geology, the true extent of the sea-spray effect is difficult to estimate. The terrestrial baseline may theoretically be highly negative but dampened by various proportions of marine sulfates.

**Fig 6:**
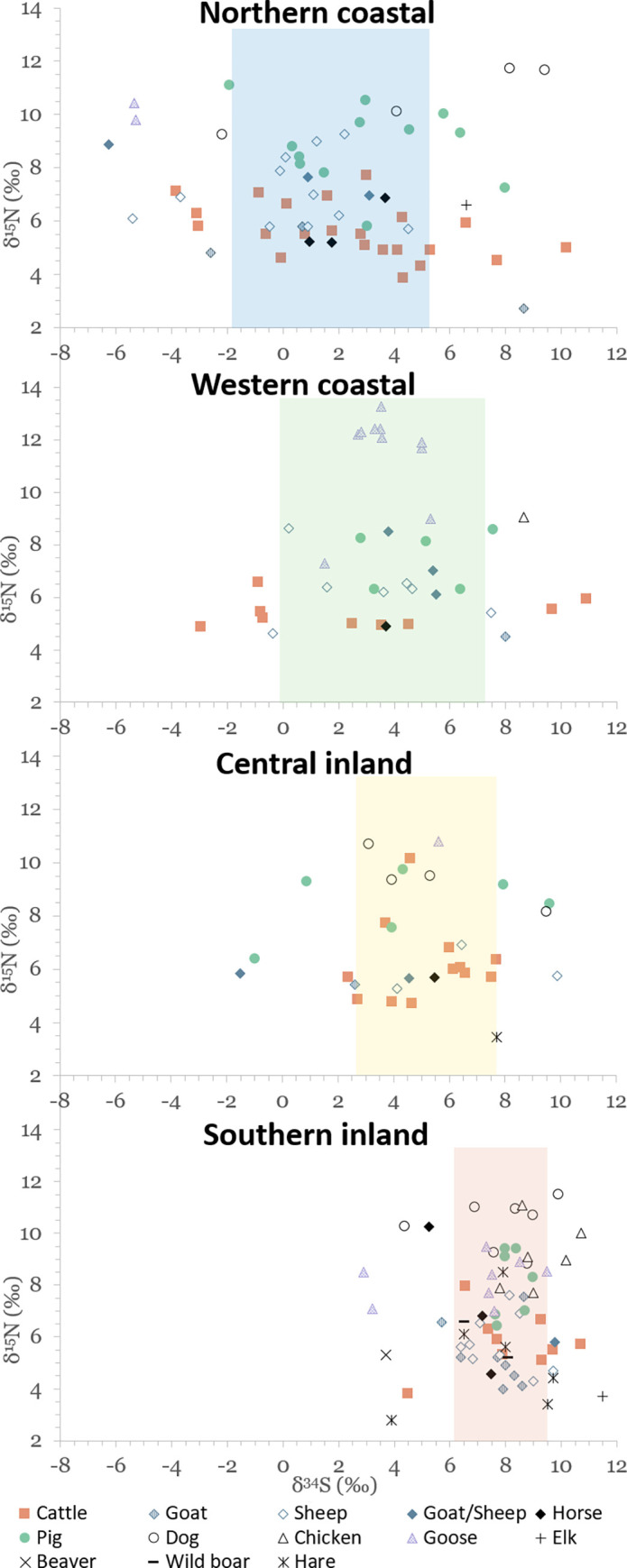
δ^34^S values of all terrestrial fauna by region. Color-shaded areas represent the 1SD range of the regional average calculated based on all terrestrial fauna.

Looking at individual terrestrial faunal δ^34^S values by region (Fig 6), ‘northern coastal’ displays the largest range of values (16.5‰), from –6.3‰ to 10.2‰. Most herbivores have δ^34^S values within the 1SD range of the regional average, however, many samples fall outside this range, including an elk (with a δ^34^S value of 6.6‰) and both herbivorous and omnivorous domesticates. It is noteworthy that two dogs with clear signals of marine resource consumption (with δ^15^N values around 12‰ and δ^13^C values around –18‰) also have some of the highest δ^34^S values in this region. This can be compared to another dog sample from the same context that displays much more ‘terrestrial’ isotopic values (9.2‰ and –20.6‰ for δ^15^N and δ^13^C, respectively), coupled with a very low δ^34^S value of –2.2‰. Based on the size of the sampled bone, this individual may have also been a wolf. It would thus seem that the lower end of δ^34^S values reported for ‘northern coastal’ may actually represent the local terrestrial baseline, whereas higher δ^34^S values can be explained by the influences of the sea-spray effect (for herbivores and omnivores) and consumption of aquatic resources (for omnivores).

Terrestrial fauna from the ‘western coastal’ region displays equally marked variation in δ^34^S values, albeit with a slightly smaller range compared to the northern coast ‒ 13.8‰, from –2.9‰ to 10.9‰. There are also notably less samples that fall outside the 1SD range for ‘western coastal’. A few domestic herbivores have δ^34^S values below 0‰. These animals may have been herded in areas closer to the northern coastline. On the other hand, herbivores with the highest δ^34^S values were likely raised on or near coastal soils influenced by the sea-spray effect (although it is also possible these animals originated from more southern/inland parts of the land). Interestingly, several geese (sampled from a single context) all have very similar δ^34^S values (furthermore, their δ^13^C and δ^15^N values are also very much alike). These particular geese were probably domestic and kept for food since their high δ^15^N values and large bone size were indicative of fattening [65]. It seems likely that these birds were kept locally and possibly on a controlled diet (i.e. not left to graze freely) so that their uniform δ^34^S values may be considered a good representation of the local baseline (possibly with a small enrichment if these geese were also consuming aquatic plants or fed fish offal).

In ‘central inland’, δ^34^S values again show a rather substantial range (11.4‰, from –1.5‰ to 9.9‰) but the results are much more concentrated compared to coastal regions. A few animals with values below 0‰ are likely from areas closer to the coast, and likewise samples with very high δ^34^S values may originate from other areas (either from the coast where they were affected by sea-spray, or from further south).

For the ‘southern inland’ region, the range of δ^34^S values is narrow compared to other areas (8.6‰, from 2.9‰ to 11.5‰). Several wild hares, which likely were local specimens, were sampled here and most fall within the 1SD range of the regional average, including the outlier with atypical carbon and nitrogen isotope values. A few herbivores have δ^34^S values around or above 10‰ which may suggest that the local terrestrial baseline might be even more positive further south/southeast. The highest reported δ^34^S value (of any terrestrial animal in this study) is from an elk (11.5‰). On the other hand, a beaver has a rather low δ^34^S value of 3.7‰, similar to two geese and a few other samples with δ^34^S values below 5‰. Fauna with such low δ^34^S values was probably non-local, brought to the ‘southern inland’ region through trade or hunting activities.

**Fig 7:**
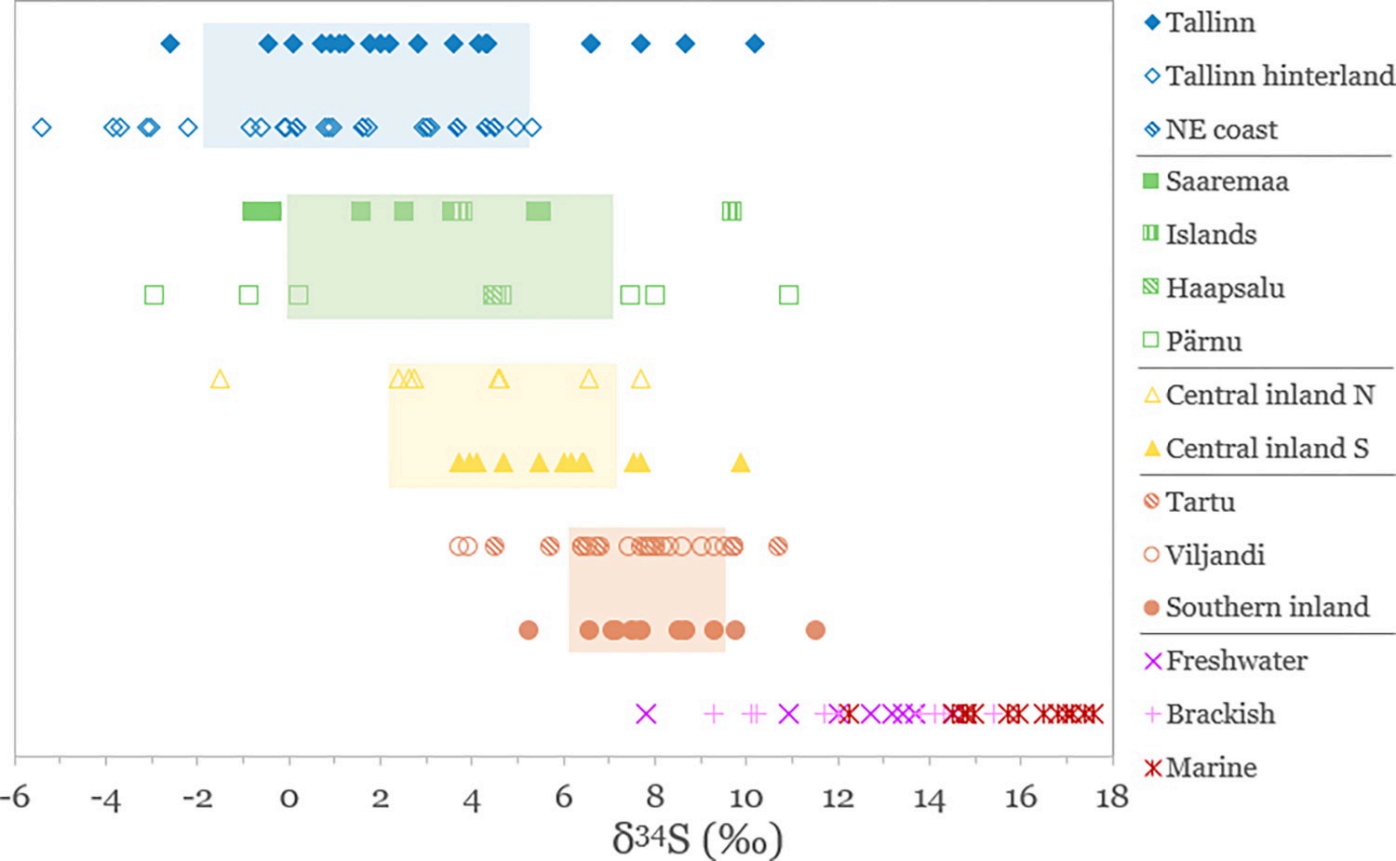
δ^34^S values of terrestrial herbivores. Samples sorted by site or smaller regions (if not enough samples from one site). Shaded rectangles mark the 1SD range of the herbivore average for each region (northern coastal ‒ blue, western coastal ‒ green, central inland ‒ yellow, southern inland ‒ red). Aquatic species included for reference.

**Fig 8:**
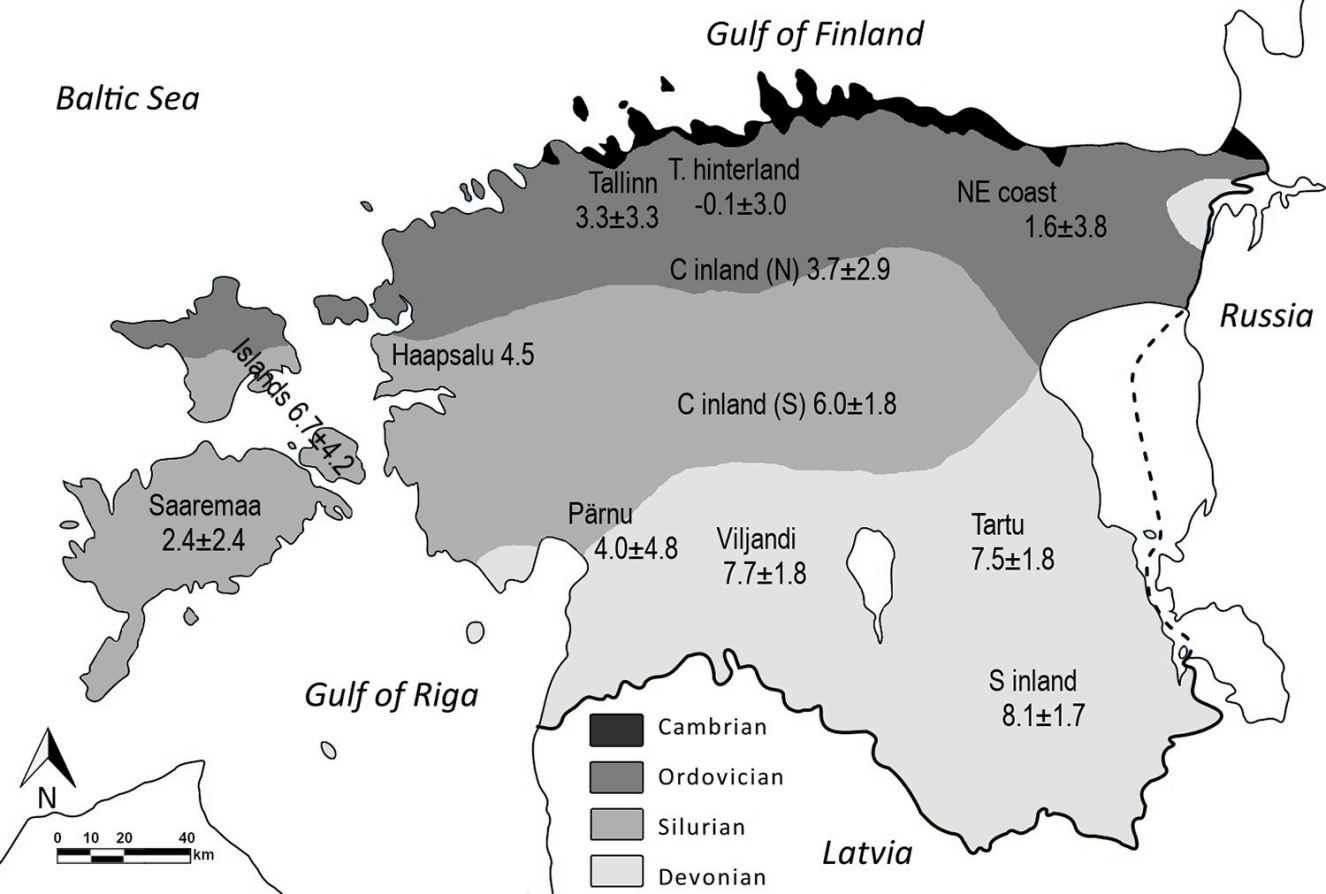
Regional sulfur isotope variation of terrestrial herbivore values on the territory of Estonia.

In Fig 7, δ^34^S values of herbivores (both wild and domestic) are compared based on site or local region (if too few samples from one site), with the 1SD range of the herbivore average for each of the four main regions marked with a rectangle. This graph is supported by Fig 8 which additionally shows average δ^34^S values plotted on the map of Estonia based on sampling area. Even though bedrock is thought to be only one of many components influencing the δ^34^S baseline of a certain region, it is clear from our data that geology seems to be a major factor behind the observed sulfur isotopic variation in this region. Further studies on rock, soil and plant δ^34^S values would be beneficial in supporting this hypothesis.

The fact that there exists such a gradient in local terrestrial faunal sulfur isotope ratios from the north-west to south-east can be extremely useful for studies of human migration and animal trade. However, this assumes that we understand all the factors influencing terrestrial faunal sulfur isotope values and can determine the local baseline with as much accuracy as possible.

The exact range of δ^34^S terrestrial baselines in coastal regions requires additional research, as the current variation among sampled herbivores is far too great and cannot be reliably used as an indicator for sulfur isotope values of local plants. However, it is reasonable to assume that the lowest reported δ^34^S values from coastal regions likely reflect the local terrestrial baseline and the remaining variation can be explained by marine influences (i.e. sea-spray). We do not know the true extent of the sea-spray effect, but in the ‘northern coastal’ region most animals with δ^34^S values below 0‰ originate from sites to the south and east of Tallinn which can be considered to represent the rural hinterland, whereas herbivores from the urban center of Tallinn have an average value of 3.3‰. In fact, all samples from the ‘northern coastal’ region with δ^34^S values above 6‰ are from Tallinn, a town situated right on the coastline.

A similar situation can be observed in the ‘western coastal’ region where most fauna with high (above 6‰) δ^34^S values are from another coastal town ‒ Pärnu. It is very likely that livestock from Tallinn and Pärnu were sometimes herded on coastal meadows (a practice that is also known from recent historical periods [86,87]) which would have been enriched with marine sulfates.

In the context of using sulfur isotope analysis to reconstruct diet and origin of humans from these periods, average δ^34^S values specific to towns and smaller regions shown in Fig 8 can be used as a reference. The local animal range (i.e. the terrestrial baseline) can be defined based on their average δ^34^S values ± 1SD. The 1-sigma standard deviation is much higher for coastal fauna but we do not believe this needs correcting since animals herded on coastal soils (with enriched δ^34^S values) were also routinely consumed and thus the larger variation would also be reflected in local human δ^34^S values. This has significant implications for using δ^34^S for palaeodietary reconstructions in this region, especially in terms of marine resource consumption, as it would be difficult to fully distinguish whether increased δ^34^S values of humans are due to consumption of aquatic resources or terrestrial resources influenced by the sea-spray effect.

For samples from ‘central inland’ region, a distinction should be made between northern and southern sites (broadly corresponding with the Ordovician/Silurian boundary shown in Fig 8) when using faunal data for local baseline reference. Northern central sites have lower δ^34^S values (on average 3.7‰) compared to southern central sites (6.0‰), although the difference is not statistically significant (Mann‒Whitney U test, U = 70.5, p = 0.082).

The fact that terrestrial fauna in the ‘southern inland’ region has high δ^34^S values approaching those seen in freshwater and marine environments, means that this proxy cannot be used to detect aquatic resource consumption in the region. However, non-local animals and migrants from northern and western regions are much more likely to be identified here, as their low δ^34^S values will appear as outliers.

### Isotopic variation of aquatic species in and around the Baltic Sea

**Fig 9:**
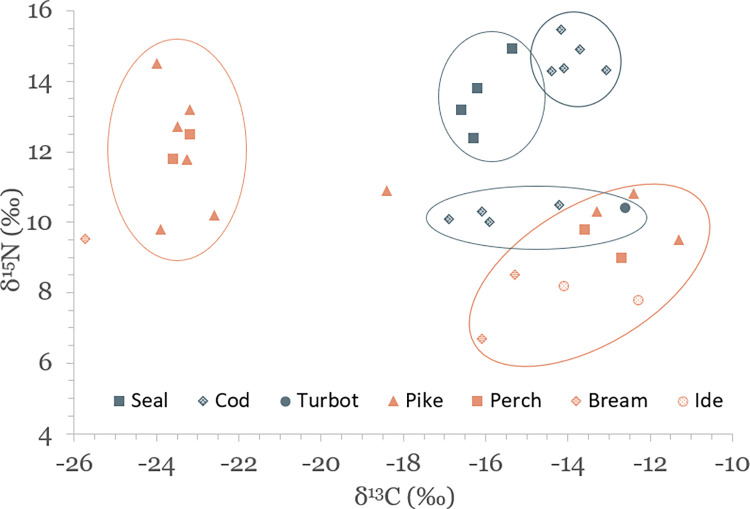
Scatterplot of δ^13^C and δ^15^N values of aquatic species sampled for this study. Gray symbols are marine species, orange symbols are freshwater. Ellipses signify niches.

The results of this study display significant variation in δ^13^C, δ^15^N and δ^34^S values of aquatic species, with seals having average values of –16.1 ± 0.5‰, 13.6 ± 1.1‰ and 16.5 ± 1.2‰, marine fish –14.5 ± 1.4‰, 12.5 ± 2.4‰ and 15.9 ± 1.5‰, and freshwater fish –18.6 ± 5.2‰, 10.4 ± 2.0‰, and 12.4 ± 2.0‰, respectively (see Table 2 and Figs 3 and 5). Marine and freshwater species are statistically significantly different in their mean δ^15^N and δ^34^S values (p<0.004 for both), but not for δ^13^C (S4 Appendix). Such a wide variation suggests that fish from a wide range of habitats were exploited for food. These potential habitats are indicated in Fig 9, where δ^13^C and δ^15^N values of analyzed species group to form distinct niches.

Five cod samples (all of them from large sized individuals) are clearly separate from the rest of the fish, displaying a ‘typical’ marine signal with δ^13^C values around –14‰ and δ^15^N values around 14‰. The combination of their size and isotope values indicates that these fish were most likely imported from the North Sea [16]. The remaining cod samples have highly variable δ^13^C values (from –14‰ to –17‰) coupled with relatively low δ^15^N values (ca. 10‰). We presume these fish to be caught locally, i.e. to represent the isotopic signal of the Baltic Sea. This would be in accordance with previously published data [16,88–90] that showed Medieval cod originating from the eastern Baltic (specifically from sites in Poland and eastern Sweden) have δ^13^C values around –16‰ to –18‰, whereas cod from western Baltic and the North Sea have carbon isotopic values around –13‰ to –15‰. Since carbon isotope ratios are directly correlated with water salinity [32], various marine consumers from the brackish Baltic Sea have been previously shown to have more negative δ^13^C values compared to ‘typical’ marine environments [14,15,91].

Cod caught from the Baltic Sea *vs*. from the North Sea are distinguished by both their δ^13^C and δ^15^N values. While the former variation is due to the brackish conditions (i.e. decreased salinity) of the Baltic Sea, lower δ^15^N values of locally caught cod may be an indication of their trophic position. North Sea cod have higher δ^15^N values because of the increased number of trophic levels in the oceanic food web. They also have faster growth rate, resulting in oceanic cod being generally larger in size [92,93]. Since the size of the fish is also closely associated with trophic levels, it is thus correlated with δ^15^N values [94]. Among the four cod specimens from the Baltic Sea, two are identified as medium-sized and two as large (when compared with local reference individuals), even though they all have relatively uniform δ^15^N values.

Two marine samples in our study (a cod and a turbot) display a combination of low δ^15^N values (resembling cod from the Baltic Sea) and high δ^13^C values (similar to cod from the North Sea). In these instances, the δ^13^C values probably do not reflect variations in seawater salinity, but rather changes in water turbidity. For example, δ^13^C of aquatic species varies based on whether they are from faster moving open water areas (offshore) or from slower moving waters (nearshore), so that very high δ^13^C values are often seen in fish living in stale waters [94]. Turbot, in fact, is a fish that prefers shallow waters near the shore [95], whereas cod are also known to sometimes favor coastal, benthic habitats [96].

Seals sampled in our study have generally uniform isotope values, suggestive of feeding on local (i.e. eastern Baltic) marine fish, except for the harp seal from Rebala (*KIA-55657*) which has higher δ^13^C and δ^15^N values (–15.4‰ and 14.9‰, respectively) compared to the other three seals (gray and ringed seals). This range of values is similar to those of imported cod and is somewhat higher than reported for Bronze and Iron Age harp seals that were feeding and breeding in the Baltic Sea [97]. However, the differences are not that significant to claim that our harp seal was necessarily non-local (i.e. migratory).

For freshwater fish, which are characterized by even greater variation in both their δ^13^C and δ^15^N values as compared to marine species, two distinct niches are clearly visible in Fig 8. Carbon isotopic variation in freshwater fish is common for species that are flexible in their occupying niches and that can live in both fast flowing riverine, nearshore, and offshore waters [94]. Variability in fish δ^15^N values, however, can be influenced by fish size but also by environmental factors such as oxygenation, pH, and nitrogen limitation (e.g., influences of wetland and upstream environments, human activities, etc.) [94].

One of the two niches (including pike, perch and bream) is characterized by carbon isotopic values typically associated with freshwater fish, with low δ^13^C values (from –23‰ to –25‰) and δ^15^N values between 10‰ and 14‰. These fish probably originated from fast flowing rivers in inland regions. The noticeable variation in δ^15^N values even within one species (e.g., pike or perch) may reflect individual differences in size. While fish samples that were identified as having belonged to large individuals had higher than average δ^15^N values, high nitrogen isotope values were also seen in those specimens that were identified as medium-sized. Most freshwater species with low carbon isotope values are from inland sites.

The other group of freshwater fish is set apart by their high δ^13^C values (up to –11‰). These include pike, perch, bream and ide. The carbon and nitrogen isotope values for this group partially overlap with those from locally caught cod (the two groups are statistically similar, p>0.08 for both), suggesting that these samples represent freshwater fish living in the brackish conditions of the Baltic Sea. The highest δ^13^C values are probably influenced by a littoral habitat, i.e. living in shallow waters close to the shore, as also suggested by Eriksson *et al*. [15], who reported very high δ^13^C (between –13‰ and –10‰) values for pike and perch from the Baltic Sea island of Öland (Sweden). The low δ^15^N values of species such as bream and ide reflect their feeding habits and lower trophic position compared to piscivorous species such as pike and perch. Most freshwater species with carbon isotope values higher than –20‰ are from coastal sites.

**Fig 10:**
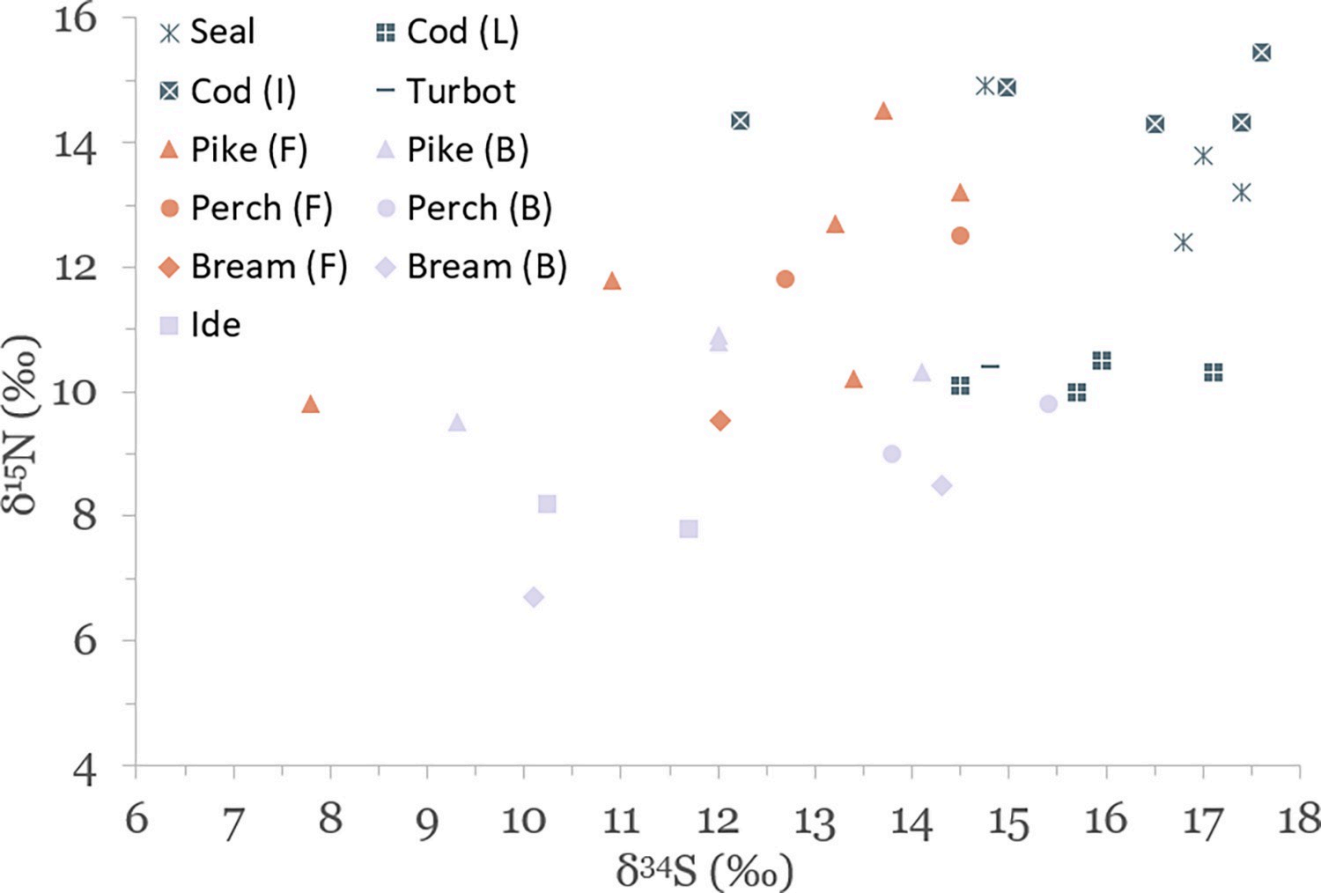
Scatterplot of δ^15^N and δ^34^S values of aquatic species sampled for this study. The distinction between ‘freshwater’ (F, orange symbols) and ‘brackish’ (B, lavender symbols) for freshwater species is made based on their δ^13^C values. L = local (Baltic Sea) cod, I = imported (North Sea) cod.

**Fig 11:**
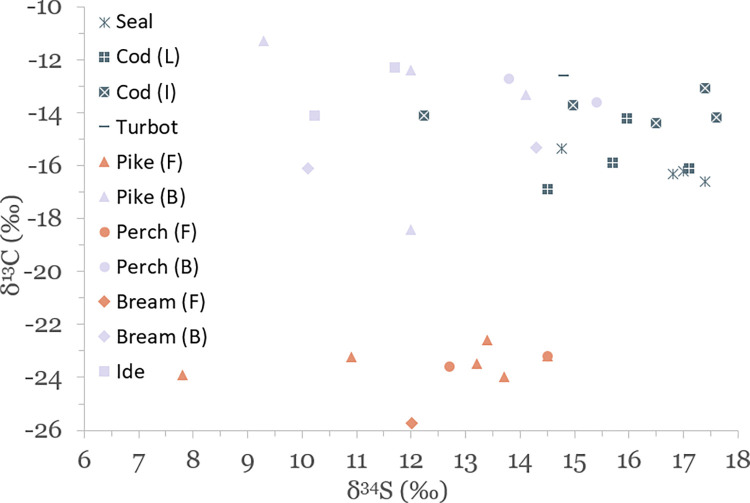
Scatterplot of δ^13^C and δ^34^S values of aquatic species sampled for this study. The distinction between ‘freshwater’ (F, orange symbols) and ‘brackish’ (B, lavender symbols) for freshwater species is made based on their δ^13^C values. L = local (Baltic Sea) cod, I = imported (North Sea) cod.

Figs 10 and 11 show δ^34^S values of aquatic species plotted against their respective δ^13^C and δ^15^N values. Marine species in our study are well distinguished by their δ^34^S values, generally falling between 15‰ and 18‰. This range of δ^34^S values was also measured for modern cod caught from the Baltic Sea [98]. A cutoff point of 15‰ is generally used for δ^34^S to distinguish between fully marine and coastal environments, with another cutoff point of 10‰ to signify marine environments with substantial riverine freshwater influx such as estuaries [14,98–100].

Interestingly, variation in δ^13^C and δ^15^N values between cod from the Baltic Sea and North Sea is not reflected in their δ^34^S values. While this may be an effect of the small sample size, it seems that marine δ^34^S values are not correlated with salinity the same way that δ^13^C values are, or that there are other factors behind the observed variability. The latter seems probable as even among cod thought to be imported from the North Sea or beyond (with relatively uniform δ^13^C and δ^15^N values) there is noticeable variation in δ^34^S values, from 12.2‰ to 17.6‰. The Rebala harp seal with isotope values similar to imported cod also has a δ^34^S value not reflective of the ‘typical’ marine sulfur isotope range (14.8‰), while the other seals believed to be local have much higher values (from 16.8‰ to 17.4‰).

The apparent overlap in oceanic *vs*. Baltic Sea δ^34^S values is all the more surprising considering that local freshwater fish living in brackish conditions have statistically significantly different δ^34^S values (but statistically similar δ^13^C) compared to purely marine species from the Baltic. While this may again be affected by the size of our dataset, the majority of brackish freshwater fish have δ^34^S values in the range of 10‰ to 15‰. This suggests that the freshwater fish mostly occupied coastal waters whereas cod and seals preferred offshore areas unaffected by freshwater sulfates. This may also explain why some of the specimens most depleted in ^34^S are also the most enriched in δ^13^C, as both of these instances occur in near-shore waters which likely experienced significant mixing of marine sulfates with freshwater and terrestrial sulfur sources.

When Leakey *et al*. [100] compared δ^34^S values of fish caught from the Thames estuary to those from adjacent coastal regions in southeast England, they observed a clear gradient of increasing δ^13^C and δ^34^S values that resulted from the mixing of marine and freshwater, with δ^34^S values ranging from –5‰ to 15‰ across the gradient. In our dataset, no similar gradient is evident when comparing freshwater species from purely freshwater (e.g., riverine) to brackish (coastal) waters. In fact, ‘freshwater’ and ‘brackish’ fish show significant overlap and no statistical differences in their respective δ^34^S values (Figs 10 and 11).

On the other hand, freshwater fish from inland locations have been shown to have highly variable δ^34^S values from site-to-site, even within one region [18,20,21]. It is possible that the observed similarity between purely freshwater and brackish fish δ^34^S values is coincidental and that the samples reported here reflect local riverine sulfates of various bodies of water. More research on fish from well-defined, single contexts in Estonia is necessary to further explore the variation in the local aquatic baselines, as there is a distinct lack of paired δ^13^C, δ^15^N and δ^34^S isotope data from the Baltic region.

## Conclusions

The large dataset analyzed here has established faunal baseline ranges to better understand the reasons behind the observed large variation in isotope values and to aid in future (human) paleodietary research of material from related contexts. Our results demonstrate that domestic omnivores and herbivores are well distinguished by their δ^13^C and δ^15^N values, although there is a great deal of intraspecies variability. No regional trends in carbon and nitrogen isotope values are evident, however, the significant variation in domestic herbivore δ^15^N values require further research to determine the exact extent and significance of the observed pattern.

This study has demonstrated the importance of sulfur stable isotope analysis in local archaeological research by showing clear differences in marine *vs*. terrestrial δ^34^S values and the existence of a north-west/south-east gradient in terrestrial δ^34^S values influenced by local geology. Low terrestrial δ^34^S values (ca. 0‰) can be interpreted as originating from coastal regions (especially the northern part of the land), whereas highly positive δ^34^S values can be caused by various factors such as aquatic resource consumption, marine sulfate influence on coastal soils, or originating from the southern inland regions. These differences can potentially be exploited to study migration at a local level, e.g., to shed more light on exogamous practices that were common in (pre)history.

Although some researchers have successfully used sulfur isotope analyses to identify freshwater resource consumption, this is problematic in our study area due to δ^34^S values of freshwater fish partly overlapping with both terrestrial and marine sulfur isotopic ranges. However, δ^13^C values of freshwater species seem to be a good indicator of their habitat, e.g., whether they originated from inland (e.g., riverine) or coastal (e.g., estuary) regions. Since aquatic resources with such diverse isotopic signals were all routinely consumed, identifying their respective signals in consumer (i.e. human) isotope values is going to be challenging.

Future studies should take advantage of the regional distribution in δ^34^S values in the territory of Estonia, supporting this with other isotopic proxies (δ^13^C and δ^15^N from bone and dentine collagen but also δ^13^C, δ^18^O and ^87^Sr/^86^Sr from bone carbonate and tooth enamel) to help distinguish between sulfur isotopic variation caused by origin and that caused by marine influence.

## Supporting information

S1 Fig Carbon and nitrogen isotope data for domestic herbivores from urban and rural contexts. (TIF)

S2 Fig Domestic herbivore carbon and nitrogen isotope ratios plotted according to assigned period and species. (TIF)

S3 Fig Scatterplot of stable sulfur and nitrogen isotope data for faunal specimens. (TIF)

S1 Appendix Full analytical results and sample context. (XLSX)

S2 Appendix ZooMS results. (XLSX)

S3 Appendix Methodological description. (DOCX)

S4 Appendix Statistical analyses. (XLSX)

## References

[1] Tõrv M, Meadows J. Radiocarbon dates and stable isotope data from the Early Bronze Age burials in Riigiküla I and Kivisaare settlement sites, Estonia. Radiocarbon. 2015;57(4):645–656.

[2] Oras E, Lang V, Rannamäe E, Varul L, Konsa M, Limbo-Simovart J, et al. Tracing prehistoric migration: isotope analysis of Bronze and Pre-Roman Iron Age coastal burials in Estonia. Est J Archaeol. 2016 Jun 1;20(1), 3–32.

[3] Oras E, Tõrv M, Jonuks T, Malve M, Radini A, Isaksson S, et al. Social food here and hereafter: Multiproxy analysis of gender-specific food consumption in conversion period inhumation cemetery at Kukruse, NE-Estonia. J Archaeol Sci. 2018 Sep 1;97:90–101.

[4] Tõrv M. Persistent practices: a multi-disciplinary study of hunter-gatherer mortuary remains from c. 6500–2600 cal. BC, Estonia. In: Hartz S, Lübke H, editors. Untersuchungen und Materialien zur Steinzeit in Schleswig-Holstein und im Ostseeraum, 9. Wachholtz Verlag GmbH: 2018.

[5] Aguraiuja-Lätti Ü, Lõugas L. Stable isotope evidence for medieval diet in urban and rural northern Estonia. J Archaeol Sci Rep. 2019 Aug 1;26:101901.

[6] Lightfoot E, Naum M, Kadakas V, Russow E. The influence of social status and ethnicity on diet in Mediaeval Tallinn as seen through stable isotope analysis. Est J Archaeol. 2016 Jun 1;20(1):81–107.

[7] Põltsam-Jürjo I. Pidusöögist näljahädani: söömine-joomine keskaja Tallinnas. Hea Lugu; 2013.

[8] Põltsam-Jürjo I. Kala tähtsusest kaubanduses, majanduses ning toidumenüüs 13.–16. sajandi Eestis. Acta Hist Tallinnensi. 2018(24):3–23.

[9] Müldner G, Richards MP. Fast or feast: reconstructing diet in later medieval England by stable isotope analysis. J Archaeol Sci. 2005 Jan 1;32(1):39–48.

[10] Linderholm A, Jonson CH, Svensk O, Lidén K. Diet and status in Birka: stable isotopes and grave goods compared. Antiquity. 2008 Jun;82:446–461.

[11] Grupe G, Heinrich D, Peters J. A brackish water aquatic foodweb: trophic levels and salinity gradients in the Schlei fjord, Northern Germany, in Viking and medieval times. J Archaeol Sci. 2009 Oct 1;36(10):2125–2144.

[12] Kjellström A, Storå J, Possnert G, Linderholm A. Dietary patterns and social structures in Medieval Sigtuna, Sweden as reflected in stable isotope values in human skeletal remains. J Archaeol Sci. 2009 Dec 1;36(12):2689–2699.

[13] Linderholm A, Kjellström A. Stable isotope analysis of a medieval skeletal sample indicative of systemic disease from Sigtuna Sweden. J Archaeol Sci. 2011 Apr 1;38(4):925–933.

[14] Craig OE, Ross R, Andersen SH, Milner N, Bailey GN. Focus: sulphur isotope variation in archaeological marine fauna from northern Europe. J Archaeol Sci. 2006 Nov 1;33(11):1642–1646.

[15] Eriksson G, Linderholm A, Fornander E, Kanstrup M, Schoultz P, Olofsson H, et al. Same island, different diet: cultural evolution of food practice on Öland, Sweden, from the Mesolithic to the Roman Period. J Anthropol Archaeol. 2008 Dec 1;27(4):520–543.

[16] Orton DC, Makowiecki D, de Roo T, Johnstone C, Harland J, Jonsson L, et al. Stable isotope evidence for Late Medieval (14th–15th c.) origins of the eastern Baltic cod (*Gadus morhua*) fishery. PLoS ONE. 2011 Nov 15;6(11):e27568. 22110675 10.1371/journal.pone.0027568 PMC3217992

[17] Richards MP, Fuller BT, Hedges REM. Sulphur isotopic variation in ancient bone collagen from Europe: implications for human palaeodiet, residence mobility, and modern pollutant studies. Earth Planet Sci Lett. 2001 Sep 15;191(3–4):185–190.

[18] Privat KL, O’Connell TC, Hedges REM. The distinction between freshwater- and terrestrial based diets: methodological concerns and archaeological applications of sulphur stable isotope analysis. J Archaeol Sci. 2007 Aug 1;34(8):1197–1204.

[19] Vika E. Strangers in the grave? Investigating local provenance in a Greek Bronze Age mass burial using δ^34^S analysis. J Archaeol Sci. 2009 Sep 1;36(9):2024–2028.

[20] Nehlich O, Borić D, Stefanović S, Richards MP. Sulphur isotope evidence for freshwater fish consumption: a case study from the Danube Gorges, SE Europe. J Archaeol Sci. 2010 May 1;37(5):1131–1139.

[21] Nehlich O, Fuller BT, Jay M, Mora A, Nicholson RA, Smith C, et al. Application of sulphur isotope ratios to examine weaning patterns and freshwater fish consumption in Roman Oxfordshire, UK. Geochim Cosmochim Acta. 2011 Sep 1;75(17):4963–4977.

[22] Oelze VM, Koch JK, Kupke K, Nehlich O, Zäuner S, Wahl J, et al. Multi‐isotopic analysis reveals individual mobility and diet at the Early Iron Age monumental tumulus of Magdalenenberg, Germany. Am J Phys Anthropol. 2012 Jul;148(3):406–421. doi: 10.1002/ajpa.22063 22553183

[23] Sayle KL, Cook GT, Ascough PL, Hastie HR, Einarsson Á, McGovern TH, et al. Application of ^34^S analysis for elucidating terrestrial, marine and freshwater ecosystems: Evidence of animal movement/husbandry practices in an early Viking community around Lake Mývatn, Iceland. Geochim Cosmochim Acta. 2013 Nov 1;120:531–544.

[24] Sayle KL, Hamilton WD, Gestsdóttir H, Cook GT. Modelling Lake Mývatn’s freshwater reservoir effect: utilisation of the statistical program FRUITS to assist in the re-interpretation of radiocarbon dates from a cemetery at Hofstaðir, north-east Iceland. Quat Geochronol. 2016 Sep 1;36:1–11.

[25] Etu-Sihvola H, Bocherens H, Drucker DG, Junno A, Mannermaa K, Oinonen M, et al. The dIANA database–Resource for isotopic paleodietary research in the Baltic Sea area. J Archaeol Sci Rep. 2019 Apr 1;24:1003–1013.

[26] Eesti Raukas A. Loodus. [Estonia. Nature] Tallinn: Valgus; 1995.

[27] Kukk T, Kull T, Luuk O, Saar P, Mesipuu M. Eesti Taimede Levikuatlas 2020 [Atlas of the Estonian Flora 2020]. Tartu: Pärandkoosluste Kaitse Ühing; 2020.

[28] Mitchell-Jones AJ, Amori G, Bogdanowicz W, Krystufek B, Reijnders PJ et al. The atlas of European mammals. London: Academic Press; 1999.

[29] Suuroja K. The bedrock geological map of Estonia (1:400 000). Geological Survey of Estonia; 1997.

[30] Björck S. The late Quaternary development of the Baltic Sea basin. In: Assessment of climate change for the Baltic Sea Basin. Springer; 2008. pp. 398–407.

[31] Rosentau A, Bennike O, Uścinowicz S, Miotk-Szpiganowicz G. The Baltic Sea basin. In: Flemming NC, Harff J, Moura D, Burgess A, Bailey GN, editors. Submerged landscapes of the European continental shelf: Quaternary paleoenvironments. John Wiley and Sons; 2017. pp. 103–133.

[32] Emeis KC, Struck U, Blanz T, Kohly A, Voß M. Salinity changes in the central Baltic Sea (NW Europe) over the last 10 000 years. Holocene. 2003 Apr;13(3):413–423.

[33] Arold I. Eesti maastikud. Tartu Ülikooli Kirjastus; 2005.

[34] Raukas A, Teedumäe A, editors. Geology and Mineral Resources of Estonia. Tallinn: Estonian Academy Publishers; 1997.

[35] Rattas M, Kalm V. Glaciotectonic deformation patterns in Estonia. Geol Q. 2004 48(1):15–22.

[36] Schoeninger MJ, Moore KM. Bone stable isotope studies in archaeology. J World Prehist. 1992 Jun;6(2):247–296.

[37] Sealy JC. Body tissue chemistry and palaeodiet. In: Brothwell DR, Pollard AM, editors. Handbook of Archaeological Sciences. Wiley, 2001. pp. 269–279.

[38] Ambrose SH, Norr L. Isotopic composition of dietary protein and energy versus bone collagen and apatite: purified diet growth experiments. In: Lambert J, Grupe G, editors. Prehistoric Human Bone: Archaeology at the Molecular Level. Springer-Verlag: New York; 1993. pp. 1–37.

[39] Tieszen LL, Fagre T. Effect of diet quality on the isotopic composition of respiratory CO_2_, bone collagen, bioapatite and soft tissues. In: Lambert JB, Grupe G, editors. Prehistoric human bone: archaeology at the molecular level. Springer-Verlag:Berlin: 1993. pp. 121–155.

[40] van Klinken GJ, Richards MP, Hedges REM. An overview of causes for stable isotopic variations in past European human populations: environmental, ecophysiological, and cultural effects. In: Ambrose SH, Katzenberg MA, editors. Biogeochemical approaches to paleodietary analysis. Kluwer Academic Publishers: New York; 2000. pp. 39–64.

[41] Hedges REM, Stevens RE, Richards MP. Bone as a stable isotope archive for local climatic information. Quat Sci Rev. 2004 Apr 1;23:959–965.

[42] Fernandes R, Nadeau MJ, Grootes PM. Macronutrient-based model for dietary carbon routing in bone collagen and bioapatite. Archaeol Anthropol Sci. 2012 Dec;4(4):291–301.

[43] Ambrose SH, DeNiro MJ. The isotopic ecology of East African mammals. Oecologia. 1986 Jun;69(3):395–406. doi: 10.1007/BF00377062 28311342

[44] McCutchan JH, Lewis WM Jr, Kendall C, McGrath CC. Variation in trophic shift for stable isotope ratios of carbon, nitrogen, and sulphur. Oikos. 2003 Aug;102(2):378–390.

[45] Reitsema LJ, Kozłowski T, Makowiecki D. Human–environment interactions in medieval Poland: a perspective from the analysis of faunal stable isotope ratios. J Archaeol Sci. 2013 Oct 1;40(10):3636–46.

[46] Simčenka E, Jakulis M, Kozakaitė J, Piličiauskienė G, Lidén K. Isotopic dietary patterns of monks: results from stable isotope analyses of a seventeenth–eighteenth century Basilian monastic community in Vilnius, Lithuania. Archaeol Anthropol Sci. 2020 May;12(5):1–4.

[47] Bocherens H, Drucker D. Trophic level isotopic enrichment of carbon and nitrogen in bone collagen: case studies from recent and ancient terrestrial ecosystems. Int J Osteoarchaeol. 2003 Jan;13:46–53.

[48] Hedges REM, Reynard L. Nitrogen isotopes and the trophic level of humans in archaeology. J Archaeol Sci. 2007 Aug 1;34(8):1240–1251.

[49] Amundson R, Austin AT, Schuur EA, Yoo K, Matzek V, Kendall C, et al. Global patterns of the isotopic composition of soil and plant nitrogen. Global biogeochem cycles. 2003 Mar;17(1):31–1.

[50] Richards MP, Hedges RE. Variations in bone collagen δ^13^C and δ^15^N values of fauna from Northwest Europe over the last 40 000 years. Palaeogeogr Palaeoclimatol Palaeoecol. 2003 Apr 15;193(2):261–7.

[51] Fraser RA, Bogaard A, Heaton T, Charles M, Jones G, Christensen BT, et al. Manuring and stable isotope ratios in cereals and pulses: towards a new archaeobotanical approach to the inference of land use and dietary practices. J Archaeol Sci. 2011 Oct 1;38(10):2790–2804.

[52] Bogaard A, Fraser R, Heaton THE, Wallace M, Vaiglova P, Charles M, et al. Crop manuring and intensive land management by Europe’s first farmers. PNAS. 2013 Jul 30;110(31): 12589–12594. doi: 10.1073/pnas.1305918110 23858458 PMC3732975

[53] Szpak P. Complexities of nitrogen isotope biogeochemistry in plant-soil systems: implications for the study of ancient agricultural and animal management practices. Front Plant Sci. 2014 Jun 23;5:288. doi: 10.3389/fpls.2014.00288 25002865 PMC4066317

[54] Jenkins SG, Partridge ST, Stephenson TR, Farley SD, Robbins CT. Nitrogen and carbon isotope fractionation between mothers, neonates, and nursing offspring. Oecologia. 2001 Nov;129(3):336–41. doi: 10.1007/s004420100755 28547188

[55] Koehler G, Kardynal KJ, Hobson KA. Geographical assignment of polar bears using multi-element isoscapes. Sci Rep. 2019 Jun 28;9(1):1–9. 31253845 10.1038/s41598-019-45874-w PMC6599000

[56] Cherney MD, Fisher DC, Hren MT, Shirley EA. Stable isotope records of nursing and weaning: A case study in elephants with implications for paleobiological investigations. Palaeogeogr Palaeoclimatol Palaeoecol. 2021 Apr 1;567:110223.

[57] Chilvers BL. Isotope values from milk and blood serum in New Zealand sea lions: are pups feeding on milk a trophic level higher than their mothers? Mar Biol. 2021 Jan;168(1):1–7.

[58] Krouse HR, Legge AH, Brown HM. Sulphur gas emissions in the boreal forest–the West Whitecourt case study V. Stable sulphur isotopes. Water Air Soil Poll. 1984 Apr;22(3):321–347.

[59] Peterson BJ, Fry B. Stable isotopes in ecosystem studies. Annu Rev Ecol Syst. 1987 Jan 1;18:293–320.

[60] Trust BA, Fry B. Stable sulphur isotopes in plants: a review. Plant Cell Environ. 1992 Dec;15(9):1105–1110.

[61] Richards MP, Fuller BT, Sponheimer M, Robinson T, Ayliffe L. Sulphur isotopes in palaeodietary studies: a review and results from a controlled feeding experiment. Int J Osteoarchaeol. 2003 Jan;13(1–2):37–45.

[62] Rees CE, Jenkins WJ, Monster J. The sulphur isotopic composition of ocean water sulphate. Geochim Cosmochim Acta. 1978 Apr 1;42(4):377–381.

[63] Wadleigh MA, Schwarcz HP, Kramer JR. Sulphur isotope tests of seasalt correction factors in precipitation: Nova Scotia, Canada. Water Air Soil Poll. 1994 Sep;77(1):1–16.

[64] Sparks JM, Crowley BE, Rutherford MG, Jaggernauth D. Coastal proximity, orientation, and precipitation amount drive spatial variability in δ^34^S values on the Caribbean island of Trinidad. Appl Geochem. 2019 Sep 1;108:104372.

[65] Ehrlich F, Aguraiuja-Lätti Ü, Lõugas L, Rannamäe E. Application of morphometric and stable isotope analyses for distinguishing domestic and wild geese. Int J Osteoarchaeol. 2022 Jan 6;32(2):457–466.

[66] Pluskowski A, Makowiecki D, Maltby M, Rannamäe E, Lõugas L, Maldre L, et al. The Baltic Crusades and ecological transformation: The zooarchaeology of conquest and cultural change in the Eastern Baltic in the second millennium AD. Quaternary International. 2019(510):28−43.

[67] Kriiska A, Lang V, Mäesalu A, Tvauri A, Valk H. Eesti ajalugu I. Eesti esiajalugu [Estonian History I. Estonian Prehistory]. Tartu Ülikooli ajaloo- ja arheoloogia instituut; 2020.

[68] van Klinken GJ. Bone collagen quality indicators for palaeodietary and radiocarbon measurements. J Archaeol Sci. 1999 Jun 1;26(6):687–695.

[69] DeNiro MJ. Postmortem preservation and alteration of *in vivo* bone collagen isotope ratios in relation to palaeodietary reconstruction. Nature 1985 Oct;317:806–809.

[70] Ambrose SH. Preparation and characterization of bone and tooth collagen for isotopic analysis. J Arch Sci. 1990 Jul 1;17:431–451.

[71] Nehlich O, Richards MP. Establishing collagen quality criteria for sulphur isotope analysis of archaeological bone collagen. Archaeol Anthropol Sci. 2009 Mar;1(1):59–75.

[72] Hedges REM, Clement JG, Thomas DL, O’Connell TC. Collagen turnover in the adult femoral mid-shaft: modeled from anthropogenic radiocarbon tracer measurements. Am J Phys Anthropol. 2007 Jun;133(2):808–816. doi: 10.1002/ajpa.20598 17405135

[73] Clark CT, Horstmann L, Misarti N. Quantifying variability in stable carbon and nitrogen isotope ratios within the skeletons of marine mammals of the suborder Caniformia. J Archaeol Sci Rep. 2017 Oct 1;15:393–400.

[74] Smith KJ, Sparks JP, Timmons ZL, Peterson MJ. Cetacean Skeletons Demonstrate Ecologically Relevant Variation in Intraskeletal Stable Isotopic Values. Front Mar Sci. 2020 Jun 3;7:388.

[75] Sykut M, Pawełczyk S, Borowik T, Pokorny B, Flajšman K, Niedziałkowska M. Intraindividual and interpopulation variability in carbon and nitrogen stable isotope ratios of bone collagen in the modern red deer (*Cervus elaphus*). J Archaeol Sci Rep. 2020 Dec 1;34:102669.

[76] Pestle WJ, Crowley BE, Weirauch MT. Quantifying inter-laboratory variability in stable isotope analysis of ancient skeletal remains. PLoS ONE. 2014 Jul 25;9(7):e102844. doi: 10.1371/journal.pone.0102844 25061843 PMC4111498

[77] Rumpelmayr K. Reconstructing diet by stable isotope analysis (δ13C and δ15N): Two case studies from Bronze Age and Early Medieval Lower Austria. PhD Thesis, Universität Wien. 2012.

[78] van der Merwe NJ, Medina E. The canopy effect, carbon isotope ratios and foodwebs in Amazonia. J Archaeol Sci. 1991 May 1;18(3):249–259.

[79] France R. Carbon isotope ratios in logged and unlogged boreal forests: examination of the potential for determining wildlife habitat use. Environ Manage. 1996 Mar;20(2):249–255.

[80] van Klinken GJ, van der Plicht H, Hedges REM. Bone ^13^C/^12^C ratios reflect (palaeo-) climatic variations. Geophys Res Lett. 1994 Mar 15;21(6):445–448.

[81] Hartman G, Danin A. Isotopic values of plants in relation to water availability in the Eastern Mediterranean region. Oecologia. 2010 Apr;162(4):837–852. doi: 10.1007/s00442-009-1514-7 19956974 PMC2841277

[82] Matthews JA, Briffa KR. The ‘Little Ice Age’: re‐evaluation of an evolving concept. Geogr Ann Ser A. 2005 Mar 1;87(1):17–36.

[83] Grove JM. The Little Ice Age. Routledge; 2012 Sep 10.

[84] Hughes MK, Diaz HF. Was there a ‘Medieval Warm Period’, and if so, where and when? Clim change. 1994 Mar;26(2):109–142.

[85] Peterson BJ, Howarth RW. Sulphur, carbon and nitrogen isotopes used to trace organic matter flow in the salt-marsh estuaries of Sapelo Island, Georgia. Limnol Oceanogr. 1987 Nov;32(6):1195–1213.

[86] Sammul M, Kull K, Kukk T. Natural grasslands in Estonia: evolution, environmental and economic roles. In: Viiralt R, editor. Conventional and Ecological Grassland Management. Comparative Research and Development. Proceedings of the International Symposium, Tartu, July 4–6, 2000. Estonian Grassland Society, 2000. pp. 20–26.

[87] Köster T, Kauer K, Tõnutare T, Kõlli R. The management of the coastal grasslands of Estonia. In: Brebbia CA, Saval Perez JM, Garcia Andion L, editors. Coastal Environment V, incorporating Oil Spill Studies. WIT Press; 2004. pp. 45−54.

[88] Orton D, Rannamäe E, Lõugas L, Makowiecki D, Hamilton-Dyer S, Pluskowski A, et al. The Teutonic Order’s role in the development of a Medieval Eastern Baltic cod fisfhery: Evidence from fish bone isotopes. In: Pluskowski A, editor. Ecologies of crusading, colonization, and religious conversion in the Medieval Baltic: Terra Sacra II. Turnhout, Belgium: Brepols Publishers (Environmental Histories of the North Atlantic World; 3); 2019. pp. 223–240.

[89] Barrett J, Johnstone C, Harland J, Van Neer W, Ervynck A, Makowiecki D, et al. Detecting the medieval cod trade: a new method and first results. J Archaeol Sci. 2008 Apr 1;35:850–61.

[90] Barrett JH, Orton D, Johnstone C, Harland J, Van Neer W, Ervynck A, et al. Interpreting the expansion of sea fishing in medieval Europe using stable isotope analysis of archaeological cod bones. J Archaeol Sci. 2011 Jul 1;38:1516–24.

[91] Fornander E, Eriksson G, Lidén K. Wild at heart: Approaching Pitted Ware identity, economy and cosmology through stable isotopes in skeletal material from the Neolithic site Korsnäs in Eastern Central Sweden. J Anthropol Archaeol. 2008 Sep 1;27(3):281–297.

[92] Link JS, Bogstad B, Sparholt H, Lilly GL. Trophic role of Atlantic cod in the ecosystem. Fish and Fisheries. 2008 Oct; 9:1–30.

[93] Neuenfeldt S, Bartolino V, Orio A, Andersen KH, Andersen NG, Niiranen S, et al. Feeding and growth of Atlantic cod (*Gadus morhua* L.) in the eastern Baltic Sea under environmental change. ICES J Mar Sci. 2020 Mar 1;77(2):624–32.

[94] Guiry E. Complexities of stable carbon and nitrogen isotope biogeochemistry in ancient freshwater ecosystems: Implications for the study of past subsistence and environmental change. Front Ecol Evol. 2019 Aug 21;7:313.

[95] Drevs T. Turbot (*Scophthalmus maximus*). In: Ojaveer E, Pihu E, Saat T, editors. Fishes of Estonia. Estonian Academy Publishers; 2003. pp. 371–373.

[96] Ojaveer E. Cod (*Gadus morhua callarias*). In: Ojaveer E, Pihu E, Saat T, editors. Fishes of Estonia. Estonian Academy Publishers; 2003. pp. 260–265.

[97] Glykou A, Lõugas L, Piličiauskienė G, Schmölcke U, Eriksson G, Lidén K. Reconstructing the ecological history of the extinct harp seal population of the Baltic Sea. Quat Sci Rev. 2021 Jan 1;251:106701.

[98] Nehlich O, Barrett JH, Richards MP. Spatial variability in sulphur isotope values of archaeological and modern cod (*Gadus morhua*). Rapid Commun Mass Spectrom. 2013 Oct 30;27(20):2255–2262. 24019191 10.1002/rcm.6682

[99] Fry B. Food web structure on Georges Bank from stable C, N, and S isotopic compositions. Limnol Oceanogr. 1988 Sep;33(5):1182–1190.

[100] Leakey CDB, Attrill MJ, Jennings S, Fitzsimons MF. Stable isotopes in juvenile marine fishes and their invertebrate prey from the Thames Estuary, UK, and adjacent coastal regions. Estuar Coast Shelf S. 2008 Apr 20;77(3):513–522.

