# Supplementary figures and images for: Multi-isotopic analysis of zooarchaeological material from Estonia (ca. 200–1800 CE): Variation among food webs and geographical regions
Source: PLoS One. 2022 Dec 27;17(12):e0279583. doi: 10.1371/journal.pone.0279583 (PMC9794088; doi:10.1371/journal.pone.0279583)

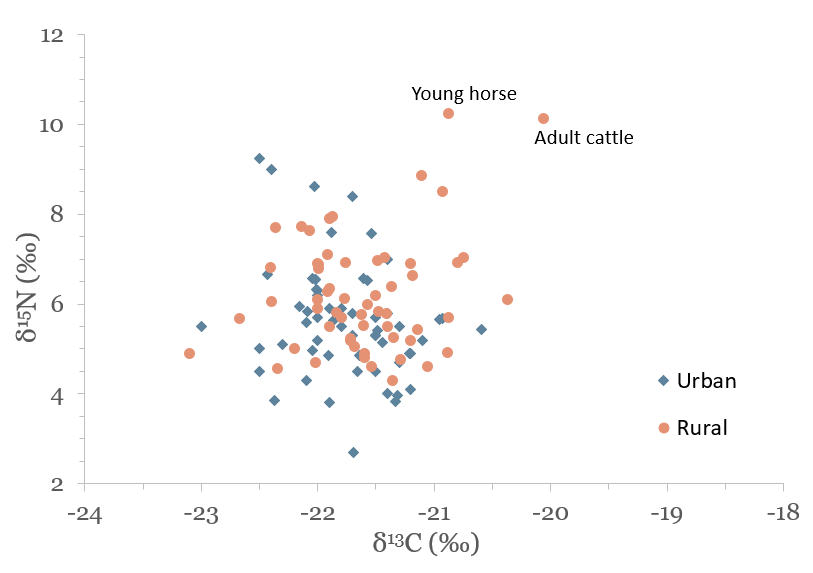

Supplement: S1 Fig — (TIF) [file pone.0279583.s001.tif]

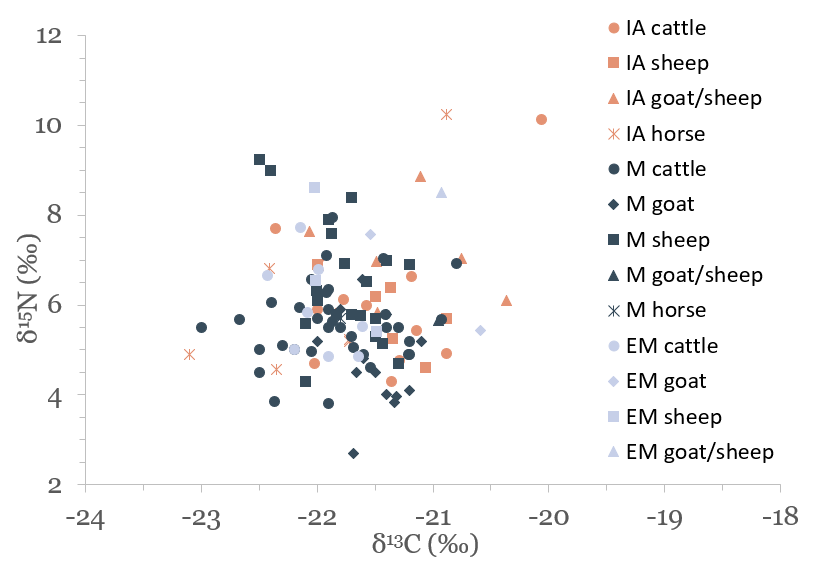

Supplement: S2 Fig — (TIF) [file pone.0279583.s002.tif]

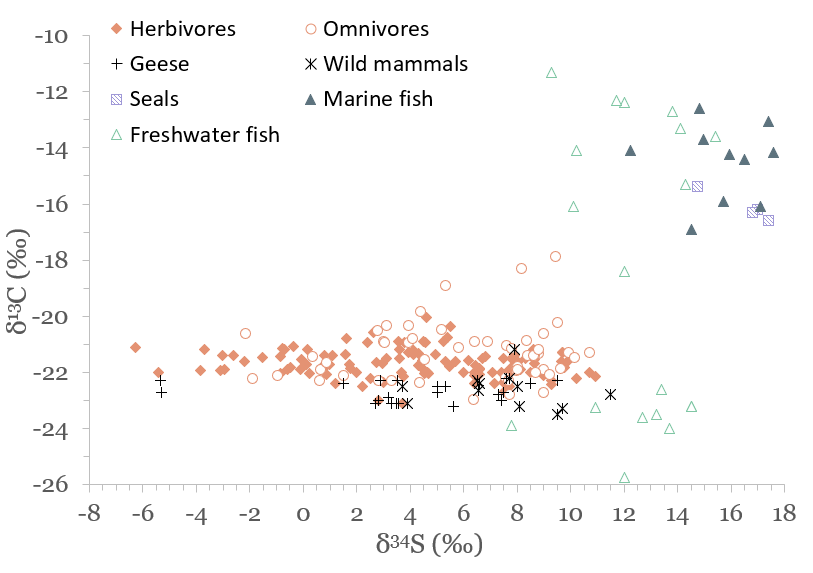

Supplement: S3 Fig — (TIF) [file pone.0279583.s003.tif]
