# Supplementary material for: Multi-isotopic analysis of zooarchaeological material from Estonia (ca. 200–1800 CE): Variation among food webs and geographical regions
Source: PLoS One. 2022 Dec 27;17(12):e0279583. doi: 10.1371/journal.pone.0279583 (PMC9794088; doi:10.1371/journal.pone.0279583)
Supplement: S3 Appendix — (DOCX) [file pone.0279583.s006.docx]

# Methodological description

For the 197 faunal bone samples from the Medieval foodways project (PRG29), collagen extraction took place in the Biochemistry Laboratory of the School of Natural Sciences and Health, at Tallinn University by the lead author (Ü. A.-L.). Pieces of approximately 0.5–1.5g of bone were cut using a Dremel multi-tool with a diamond cutting disc. All samples were physically cleaned with a sterile surgical blade removing the outer 1mm of the bone. They were then washed in ultrapure (MilliQ™) water in an ultrasonic bath for 20min and left to dry for 48h. Bone samples were demineralized in 100ml of 0.5–1M hydrochloric acid for 24–48h in room temperature. Samples were rinsed with ultrapure water and gelatinized in water at 80°C for approximately 20h. The gelatin solution was then filtered using a glass microfiber filter and freeze-dried.

Stable nitrogen (δ^15^N), carbon (δ^13^C), and sulfur (δ^34^S) isotopic compositions were determined using a Delta V Advantage continuous-flow isotope ratio mass spectrometer coupled via a ConfloIV to an IsoLink elemental analyser (Thermo Scientific, Bremen) at the Scottish Universities Environmental Research Centre (SUERC) Radiocarbon Laboratory in East Kilbride as described in Sayle *et al.* (2019). Bone collagen samples were weighed into tin capsules (~1.2–1.5mg) and combusted in the presence of oxygen in a single reactor containing tungstic oxide and copper wires at 1020°C to produce N_2_, CO_2_ and SO_2_. A magnesium perchlorate trap was used to eliminate water produced during the combustion process, and the gases were separated in a GC column heated between 70°C and 240°C. Helium was used as a carrier gas throughout the procedure. N_2_, CO_2_, and SO_2_ entered the mass spectrometer via an open split arrangement within the ConfloIV and were analyzed against their corresponding reference gases.

The International Atomic Energy Agency (IAEA) reference materials USGS40 (L-glutamic acid, δ^13^C_VPDB_ = –26.39 ± 0.04‰, δ^15^N_AIR_ = –4.52 ± 0.06‰) and USGS41a (L-glutamic acid, δ^13^C_VPDB_ = 36.55 ± 0.08‰, δ^15^N_AIR_ = 47.55 ± 0.15‰) were used to normalize δ^13^C and δ^15^N values. Two in-house standards that are calibrated to the International Atomic Energy Agency (IAEA) reference materials IAEA-S-2 (silver sulfide, δ^34^S_VCTD_ = 22.62 ± 0.08‰) and IAEA-S-3 (silver sulfide, δ^34^S_VCTD_ = –32.49 ± 0.08‰) were used to normalize δ^34^S values. Results are reported as per mil (‰) relative to the internationally accepted standards VPDB, AIR and VCDT. Normalization was checked using either the marine collagen USGS88 (^13^C_VPDB_ = –16.06 ± 0.07‰, δ^15^N_AIR_ = 14.96 ± 0.14‰, and δ^34^S_VCTD_ = 17.10 ± 0.44‰), the porcine collagen USGS89 (δ^13^C_VPDB_ = –18.13 ± 0.11‰, δ^15^N_AIR_ = 6.28 ± 0.12‰, and δ^34^S_VCTD_ = 3.86 ± 0.56‰) and/or the well characterized Elemental Microanalysis IRMS fish gelatin standard B2215 (δ^13^C_VPDB_ = –22.92 ± 0.10‰, δ^15^N_AIR_ = 4.26 ± 0.12‰, and δ^34^S_VCTD_ = 1.21 ± 0.24‰). Precision was determined to be ±0.1‰ for δ^13^C, ±0.2‰ for δ^15^N, and ±0.4‰ for δ^34^S on the basis of repeated measurements of an internal horse bone standard. Approximately 20% of samples were run in duplicate and the average 1-sigma standard deviation of the duplicates was δ^13^C: ±0.03‰, δ^15^N: ±0.03‰ and δ^34^S: ±0.3‰.

For the stable isotope values from the “Georeferenced dataset of stable carbon and nitrogen isotope values of prehistoric Estonia and its neighboring areas”, the collagen extraction was performed at the Leibniz Laboratory for Radiometric Dating and Stable Isotope Research and the EA-IRMS analyses conducted at the Isolab GmbH, Germany. For the collagen extraction an adaptation of the method described by Longin (1971) was used. First, pieces of cleaned and crushed solid bone (0.5–2mm) were demineralized in ca. 1% HCl. To remove mobile humic acids, the demineralized bone was then treated with 1% NaOH (20°C, 1h) and again with 1% HCl (20°C, 1h). Then the bone collagen was dissolved overnight as gelatin in demineralized water at 85°C and pH=3. Insoluble particles are removed by filtration through a quartz microfiber filter (DOP 0.3μm: < 0.002%). The gelatin solution was then freeze-dried. For stable isotope measurements, four collagen sub-samples, 3mg each, were sent to isolab® GmbH, Laboratorium für Stabilisotopenanalytik, for quadruplicate measurement of %C, %N, %S, atomic C:N ratio, δ^13^C, δ^15^N, and δ^34^S.

For multi-element isotope analysis of C- and N-isotopes, 3.0mg of the collagen sample were weighed into tin capsules if possible in quadruplicate. Internal standards used for calibration were casein and two different collagen standards (I: δ^13^C: –13.19 ± 0.09‰, δ^15^N: 4.55 ± 0.09‰, >50 series of measurement, and II: δ^13^C: –18.05 ± 0.06‰, δ^15^N: 5.99 ± 0.05‰, >50 series of measurement). For δ^15^N scale calibration with inorganic reference materials were performed (IAEA-NO-3 and USGS25 for δ^15^N). Scale calibration for δ^13^C was performed with organic materials NBS 22 (oil) and IRMM-BCR 657 (glucose) (Brand *et al.* 2014). Stable isotope ratios of carbon and nitrogen (δ^13^C and δ^15^N) in collagen samples were analyzed at Isolab GmbH, Schweitenkirchen, Germany, according to Sieper *et al.* (2006). Measurements were carried out using an Elemental Analyzer-Isotope-Ratio-Mass Spectrometer (EA-IRMS); for C-N-S simultaneous analysis an Elementar Pyrocube Vario Cube EL (Elementar Analysensysteme GmbH, Hanau, Germany) connected with an Isoprime Vision mass spectrometer (Isoprime Ltd. Cheadle Hulme, UK) was used. The analytical precisions using at least triplicate measurements were ±0.1‰/V-PDB for δ^13^C and ±0.2‰/ATM for δ^15^N.

For 13 goat/sheep samples that were not possible to identify to species by morphology, the species was determined using collagen peptide mass fingerprinting – Zooarchaeology by Mass Spectrometry (ZooMS) (Buckley *et al.* 2009). Work was conducted at BioArCh, Department of Archaeology, University of York (United Kingdom), following a conventional destructive ZooMS method (McGrath *et al.* 2019). Briefly, a bone sample of 15–30mg was demineralised in 0.6M hydrochloric acid, washed with 0.1M sodium hydroxide and then rinsed with 50mM ammonium bicarbonate solution (pH 8.0) three times. Then, the samples were gelatinised in ammonium bicarbonate, digested with trypsin overnight, and acidified with 5% trifluoroacetic acid solution. The peptides were extracted using C18 ZipTip® pipette tips (EMD Millipore), treated with 0.1% trifluoroacetic acid washing solution and 50% acetonitrile / 0.1% trifluoroacetic acid conditioning solution. The latter was used to elute the peptides. Samples were spotted on to a Bruker ground steel target plate and mixed with α-cyano-4-hydroxycinnamic acid matrix solution (1% in 50% acetonitrile / 0.1% trifluoroacetic acid (v/v/v)). An external calibrant was spotted adjacent to all samples, and the samples were run on Bruker Ultraflex III MALDI TOF/TOF mass spectrometer. Spectra were analyzed in mMass v.5.5 (Niedermeyer & Strohalm 2012; Strohalm *et al.* 2008, 2010) and individual peptides were identified according to previously published markers (Buckley *et al.* 2009; Kirby *et al.* 2013; Welker *et al.* 2016).

## References:

Brand WA, Coplen TB, Vogl J, Rosner M, Prohaska T. Assessment of international reference materials for isotope-ratio analysis (IUPAC Technical Report). Pure Appl Chem. 2014 Mar 20;86(3):425–467.

Buckley M, Collins M, Thomas-Oates J, Wilson J. Species identification by analysis of bone collagen using matrix-assisted laser desorption/ionisation time-of-flight mass spectrometry. Rapid Commun Mass Spectrom. 2009 Dec 15;23(23):3843–3854.

Kirby DP, Buckley M, Promise E, Trauger SA, Holdcraft TR. Identification of collagen-based materials in cultural heritage. Analyst. 2013;138(17):4849–4858.

McGrath K, Rowsell K, Gates St-Pierre C, Tedder A, Foody G, Roberts C, Speller C, Collins M. Identifying archaeological bone via non-destructive ZooMS and the materiality of symbolic expression: examples from iroquoian bone points. Sci Rep. 2019 Jul 30;9(1):1–10.

Niedermeyer THJ, Strohalm M. mMass as a software tool for the annotation of cyclic peptide tandem mass spectra. PloS ONE. 2012 7(9):e44913.

Sayle KL, Brodie CR, Cook GT, Hamilton WD. Sequential measurement of δ^15^N, δ^13^C and δ^34^S values in archaeological bone collagen at the Scottish Universities Environmental Research Centre (SUERC): a new analytical frontier. Rapid Commun Mass Spectrom. 2019 Aug 15;33(15):1258–1266.

Strohalm M, Hassman M, Košata B, Kodíček M. mMass data miner: an open source alternative for mass spectrometric data analysis. Rapid Commun Mass Spectrom. 2008 Mar 30;22(6):905–908.

Strohalm M, Kavan D, Novák P, Volný M, Havlíček V. mMass 3: a crossplatform software environment for precise analysis of mass spectrometric data. Anal Chem. 2010 Jun 1;82(11):4648–4651.

Welker F, Hajdinjak M, Talamo S, Jaouen K, Dannemann M, David F, et al. Palaeoproteomic evidence identifies archaic hominins associated with the Châtelperronian at the Grotte du Renne. Proc Natl Acad Sci. 2016 Oct 4;113(40):11162–11167.
